# Calculations of NMR properties for sI and sII clathrate hydrates of methane, ethane and propane

**DOI:** 10.1007/s00894-014-2511-2

**Published:** 2014-11-19

**Authors:** Paweł Siuda, Joanna Sadlej

**Affiliations:** Faculty of Chemistry, University of Warsaw, Pasteura 1, 02-093 Warsaw, Poland

**Keywords:** Clathrate hydrates, NMR, DFT

## Abstract

Calculations of NMR parameters (the absolute shielding constants and the spin-spin coupling constants) for 5^12^, 5^12^6^2^ and 5^12^6^4^ cages enclathrating CH_4_, C_2_H_6_ and C_3_H_8_ molecules are presented. The DFT/B3LYP/HuzIII-su3 level of theory was employed. The ^13^C shielding constants of guest molecules are close to available experimental data. In two cases (the ethane in 5^12^ and the propane in 5^12^6^2^ cages) the ^13^C shielding constants are reported for the first time. Inversion of the methyl/methylene ^13^C and ^1^H shielding constants order is found for propane in the 5^12^6^2^ cage. Topological criteria are used to interpret the changes of values of NMR parameters of water molecules and they establish a connection between single cages and bulk crystal.

**Electronic supplementary material:**

The online version of this article (doi:10.1007/s00894-014-2511-2) contains supplementary material, which is available to authorized users.

## Introduction

Clathrate hydrates are solid structures composed of a water lattice forming cages in which guest molecules reside. In these structures, the guest molecules (lower hydrocarbons, noble gases, H_2_, N_2_ etc.) engage in weak interactions with the water molecules forming cage walls of the host lattice. The most intensively studied representatives of clathrate hydrates are those encapsulating methane and carbon dioxide molecules. Abundant in nature, methane clathrate hydrate is recognized as a potential energy resource [1, 2]. Synthetic hydrates are recognized as novel materials that could be exploited for a number of practical applications, sequestration of CO_2_ being one of the most important nowadays [3–5].

The crystalline structure of the clathrate hydrates (CHs) is made up of H-bonded water molecules forming a network with cages of different shapes and sizes. The small 5^12^ cage is common to all three main structures sI, sII and sH. Small guest molecules form in general sI structures with a unit cell composed of six large 14-sided cages 5^12^6^2^ and two cages 5^12^ for a total of 46 water molecules [6]. Larger guest molecules form structure sII, with the unit cell composed of 136 water molecules (although small ones as Ar, Kr, O_2_ and N_2_ also form sII). They are made up of 24 polyhedral cages: eight large 16-sided cages 5^12^6^4^ and 16 pentagonal small 12-sided cages 5^12^. We would like to address here the structure I (sI) and structure II (sII) [7, 8] with methane, ethane and propane as guest molecules.

Spectroscopic methods with quantum chemical calculations provide a valuable tool for the study of hydrates properties from a molecular viewpoint which complement thermodynamic and kinetic studies [9–13]. Molecular vibrations of guest molecules in the clathrate hydrates (CH) vary depending on the state, on dynamics of the gas phase and on the molecular environment of the encapsulating cages. The vibrational spectra of many guest molecules in CH have been observed with IR and Raman spectroscopy. Sloan et al. [14] published the Raman spectra of CH_4_ and CO_2_ and their mixtures in cages of the hydrate clusters. The change of the frequencies and band shape upon variation of host molecules have been observed. Recently, experimental Raman spectra were published: Ohno et al. [15] found that the symmetric stretching mode in CH_4_ of 5^12^6^2^ cages were shifted to lower frequency. Complementary to experimental results density functional theory (DFT) with a dispersion correction have been employed by Ramya et al. [16] to characterize the changes in the vibrational modes of CH_4_ and H_2_O in static-type 5^12^ and larger 5^12^6^2^, explicit cages. Moreover, molecular vibrations were studied by *ab* *initio* molecular dynamics simulations with the Car-Parrinello method also by Hiratsuka et al. [17, 18] for the methane in cages 5^12^ and larger H structure 4^3^5^6^6^3^. The vibrational frequencies for methane are lower in the large cages than those in the small ones, in agreement with experimental Raman data.

NMR spectra are a valuable source of information on the molecular and the electronic structure, the conformational changes of a molecule and its environment. Chemical shifts are also widely used to understand the nature of the guest-cage interactions and dynamics of guest molecules in clathrate hydrate for several guest molecules as xenon [19, 20], methane [21–30] and many others molecules [31–34]. From the first application of NMR technique to hydrocarbon hydrate [35] to nowadays, NMR method can be used to adopted in many researches. It is the most powerful methods to elucidate the molecular properties of clathrate hydrates [36–41].

We have demonstrated the use of calculated NMR parameters for the analysis of molecular interactions of the methane and carbon dioxide with host-water molecules in static hydrates [42, 43]. As in the papers cited above, the calculations of chemical shifts (CS) and indirect spin-spin coupling constants (SSCC) (presented for the first time) showed that the environment of the encapsulating cage affects the parameters of the CH_4_ and CO_2_ molecules significantly. Recently, the experimental ^13^C chemical shift has been used for identification of ethane and propane in the 16-hedral cages of the type sII [44, 45]. The authors found experimentally that the clathrate hydration of propane reverses the ^13^C chemical shifts of methyl and methylene carbons in propane molecule as a guest to gaseous propane at room temperature and atmospheric pressure.

Pure methane is known to form sI clathrate hydrate, in which it occupies the vast majority of small 5^12^ and larger 5^12^6^2^ cages [6, 46]. Pure ethane also forms sI structure, but the occupation of cages is different than for methane. Although larger 5^12^6^2^ cages are filled with ethane, for a long time it was believed that small 5^12^ cages remain empty. However, Udachin et al. showed [47] that a few percent of small cavities are also filled with ethane molecules, which was later confirmed by Takeya et al. [48]. However, ^13^C NMR spectra recorded to date did not show signals attributable to ethane residing in the small cavity, which can be explained in terms of sensitivity of this method. Propane on its own forms a sII hydrate structure, in which bigger 5^12^6^4^ cages are occupied and smaller 5^12^ cages are left empty [6, 35, 37, 49, 50].

As natural hydrates are almost always formed by mixtures of lower hydrocarbons and other molecules, many studies were already devoted to analysis of hydrates formed by binary and ternary mixtures of CH_4_, C_2_H_6_ and C_3_H_8_. It is already known that changing concentrations of CH_4_, C_2_H_6_ and C_3_H_8_ and pressure-temperature conditions, both sI and sII structures could be obtained [51–53]. Those studies revealed, that methane and ethane could reside in all three cages forming sI and sII structures, while propane, due to its size, could be found probably only in 5^12^6^2^ and 5^12^6^4^ cages.

Those results were obtained from thermochemical or X-ray studies. The authors did not find any NMR data for ethane residing in 5^12^ cage of sI or sII structure or propane in 5^12^6^2^ cage of sI structure. Moreover, neither ^1^H shielding constants for all guest molecules of aforementioned hydrates nor spin-spin coupling constants for them could be found in the literature. Moreover, the NMR spectra were not measured due to limitations of available experimental methodology. It is expected that with the advent of more sensitive NMR experimental set-ups and methods the calculated results undertaken by us will be verified experimentally.

This work is a continuation of our previous study of water clusters [54], methane [42] and CO_2_ clathrates [43]. The cages may be perceived as clusters of water molecules interacting with hydrocarbons or as models of real CHs. Our model of CH is based on few approximations: (i) all water molecules are four coordinated (of DDAA type, double donor and double acceptor) in real crystals, while in our models all water molecules are three coordinated (of DAA and DDA types) [54]; (ii) the influence of external cages, present in the three dimensional crystalline structure of real clathrate hydrates is not considered in the model; (iii) to accurately reconstruct all features of NMR parameters in real CHs crystals, many possible structures of the guest molecules inside cages should be generated (with the use, for example, of molecular dynamics) and results weighted by the Boltzmann’s factor of energy. Instead, we are taking for each cage only one structure and the guests are assumed to be stationary; thus we ignoring the difference their crystallographic symmetries. As NMR properties do not require to operate with an optimized geometry as frequencies calculations (see the static-type calculations in [16, 17, 19]), the geometry of the cages were based on experimental X-ray data [55, 56] (as in previous paper [42]); (iv) it should be also mentioned, that many proton configurations in the cages are possible and they should be averaged as well in order to represent real hydrate in detail. But our systems are static, in the sense, that we are using single geometries for all cages; (v) the last approximation of our model addresses rovibrational effects. We do not calculate them in this work, but wherever experimental or computational data exist, they are included in the discussion of the results. Detailed description of the validity of those approximations is presented in former paper on hydrates [42]. Our aim in this work was to expand this knowledge to simple model hydrocarbons abundant in natural clathrate hydrates, in spite of the above shortcomings.

The structure of this paper is as follows: the method section employed for geometry optimization and the calculations of NMR parameters are described in Section Computational methods. Section Results and discussion presents the results of calculations and the discussion of the results. A brief summary is presented in the last section.

## Computational methods

### Geometry optimization

As a model systems we have chosen cages 5^12^ (found in sI and sII hydrate structures), 5^12^6^2^ (of sI clathrate hydrate) and 5^12^6^4^ (of sII clathrate hydrate). The locations of the water’s oxygen atoms residing in the vertices of the cages were based on X-ray data [55, 56]. As our model is static one, the starting proton arrangement in water molecules is one of possible arrangement consistent with the Bernal and Fowler ice rule [57] and the guest molecules were then inserted into cages. Other proton configurations with very different local face dipole moments are not taken into account.

We have applied a geometry optimization scheme previously used for the CO_2_ hydrates [43] and similar to one used in the former paper on the methane hydrate [42]. Thus: (i) Density-Functional Theory (DFT), using the hybrid three-parameter Becke-Lee-Yang-Parr (B3LYP) functional [58, 59] with the basis set aug-cc-pVDZ [60] was employed. (ii) During geometry optimization, the positions of the water oxygen atoms were frozen in order to preserve the overall structural characteristics of the clathrate hydrates. (iii) No counterpoise corrections were applied for the basis set superposition errors (BSSE). In the calculations we have limited ourselves to obtaining stationary geometries starting from the neutronographic structures as our cages were designed to represent structures abundant in bulk crystal. We were not interested in finding the global minima. The Gaussian03 package [61] was used to perform the geometry optimizations.

### NMR parameters calculations

According to the results of our former studies on similar systems [42, 62] which have proven to be reliable, the DFT/B3LYP approach and the HuzIII-su3 [63] basis set were chosen for the calculations of NMR parameters. To obtain accurate prediction of NMR parameters computationally, a proper description of the electron density close to the nuclei is required [64]. On the other hand, the presence of H-bonds enforces the use of diffuse functions in order to accurately describe those interactions.

It is well known that conventional DFT functionals such as B3LYP produce values that are too deshielded relative to experiment and to the best ab initio calculated values [65]. Therefore, we will focus the discussion of the results on the relative changes occurring from the complexation. Calculations were performed using the Dalton [66] package.

## Results and discussion

### Structures

**Fig. 1:**
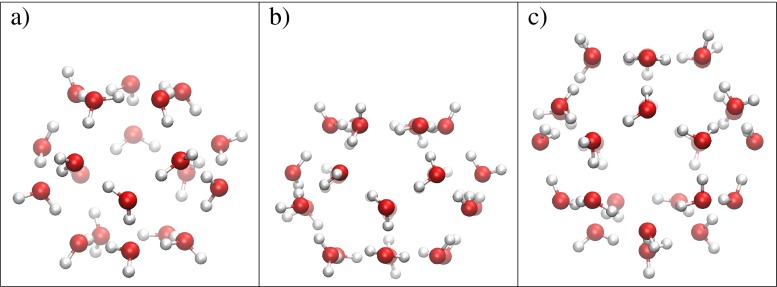
The structures of the cages: **a** 5^12^; **b** 5^12^6^2^; **c** 5^12^6^4^

The smallest clathrate cage, denoted 5^12^, consists of 20 water molecules forming 12 pentagonal faces. Cages, denoted 5^12^6^2^ and 5^12^6^4^, are built of 24 and 28 water molecules, respectively. In addition to 12 pentagonal faces they consist of two and four hexagonal faces. Stationary structures obtained for all three cages are illustrated in Fig. 1. The arrangement of a hydrogen bond network is typical to polyhedral water clusters (PWC’s) [67]. Half of the water molecules are double donor and single acceptor of protons (we denote them as DDA), while the second half donate one and accept two protons (we denote them as DAA) [54]. The DDA-type water molecules have a lone electron pair that does not accept a hydrogen bond, while the DAA-type water molecules have one OH bond not involved in hydrogen bond (dangling OH bond).

### Shielding constants of CH_4_, C_2_H_6_ and C_3_H_8_.

The ^13^C and^1^H shielding constants for encaged and gaseous guest molecules are presented in Tables 1 and 2 together with available experimental results. The ^13^C experimental data are rescaled to absolute values using recent benchmark results for pure liquid TMS (183.20 ppm) and 1 % solution of TMS in CDCl_3_ (183.94 ppm) by Jackowski and Makulski [68]. The ^1^H shielding constants are referenced to benchmark values for gaseous molecules by Garbacz et al. [69], obtained with extrapolation to zero density, giving results characterizing single, non-interacting molecules. As the interactions between CH_4_, C_2_H_6_ and C_3_H_8_ guest molecules and hydrogen-bonded water network forming cages are predominantly of dispersive character, the main factors affecting shielding constants are the size and shape of the cage. The size of the cages determines, whether guest molecules could rotate inside. Symmetry of the cavity also plays a part as it may favour distinct orientations of guest molecule in cages big enough to provide rotational freedom to the guest molecule. Different orientations of the guest could result in different shielding constants of nuclei forming quest molecule.

**Table 1:** The comparison of the calculated (B3LYP/aug-cc-pVDZ) and the experimental ^13^C shielding constants (in ppm) for the methane, the ethane and the propane molecules in the 5^12^, 5^12^6^2^ and 5^12^6^4^ cages and the gaseous state

Atom type	5^12^	5^12^6^2^	5^12^6^4^	Gaseous
**C**H_4_	187.60^*a*^ [45]	190.00^*a*^ [45]	191.50^*a*^ [45]	191.85^*a*^ [70]
	182.01^*b*^ [42]	183.28^*b*^ [42]	184.77^*b*^	188.03^*b*^ [42]
**C**_2_H_6_		175.30^*a*^ [45]	177.00^*a*^ [45]	178.35^*a*^ [71]
	164.48^*b*^	166.02^*b*^	167.14^*b*^	171.68^*b*^
C_3_H_8_:**C**H_3_			165.63^*a*^ [44]	167.22^*a*^ [44]
		156.31^*b*^	157.07^*b*^	162.18^*b*^
C_3_H_8_:**C**H_2_			166.41^*a*^ [44]	165.54^*a*^ [44]
		157.32^*b*^	156.23^*b*^	158.96^*b*^

^a^ experimental data

^b^ averaged computational data (this work)

raw data for methyl group of ethane:5^12^ 164.06, 164.89; 5^12^6^2^ 163.98, 168.05; 5^12^6^4^ 168.22, 166.07

raw data for methyl group of propane:5^12^6^2^ 157.07, 155.55; 5^12^6^4^ 156.60, 157.53

**Table 2:** The comparison of the calculated (B3LYP/aug-cc-pVDZ) and the experimental ^1^H shielding constants (in ppm) for the methane, the ethane and the propane molecules in the 5^12^, 5^12^6^2^ and 5^12^6^4^ cages and the gaseous state

Atom type	5^12^	5^12^6^2^	5^12^6^4^	Gaseous	
	Calc.	Calc.	Calc.	Calc.	Exp.
C**H**_4_	31.10 [42]	31.08 [42]	31.11	31.65 [42]	30.633 [69]
C_2_ **H**_6_	30.12	30.24	30.43	30.92	29.887 [69]
C_3_H_8_:C**H**_3_		30.17	30.29	30.94	29.832 [69]
C_3_H_8_:C**H**_2_		30.20	30.03	30.48	29.385 [69]

#### Carbon atoms

Most experimental data (the chemical shifts and the anisotropy) for clathrate hydrates containing CH_4_, C_2_H_6_ and C_3_H_8_ are recorded for ^13^C nuclei (for some mixtures with N_2_ or noble gases NMR spectra for other nuclei were also recorded).

**Fig. 2:**
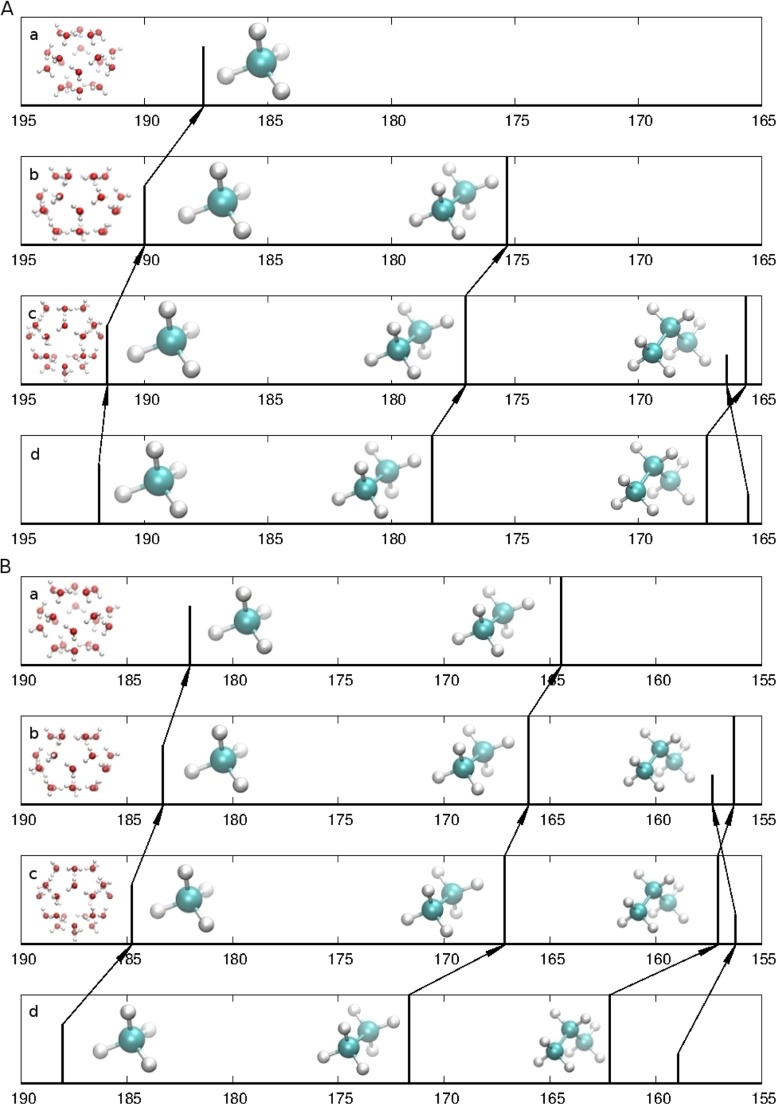
**a** The experimental^13^C absolute shielding constants for the methane, the ethane and the propane in the gas phase (lowest graph, d) and enclathrated in (a) 5^12^, (b) 5^12^6^2^ and (c) 5^12^6^4^ cages of structures sI and sII clathrate hydrates. **B** Calculated (B3LYP/aug-cc-pVDZ)^13^C absolute shielding constants for molecules in the gas phase (lowest graph, d) and enclathrated in (a) 5^12^, (b) 5^12^6^2^ and (c) 5^12^6^4^ cages of structures sI and sII clathrate hydrates

Let us first discuss shielding constants (see Table 1). Both experimental and calculated results show a monotonic trend - the ^13^C shielding constant is growing with the cage size toward the value of gaseous guest molecule (see Fig. 2 too). The changes caused by the enclathration found experimentally for methane hydrates are equal to -4.25 ppm, -1.85 ppm and -0.35 ppm [45] (for 5^12^, 5^12^6^2^ and 5^12^6^4^, respectively). The calculated values are -6.02 ppm, -4.75 ppm and -3.26 ppm [42]. The computational changes of the ^13^C chemical shielding connected with the enclathration are systematically greater than experimental ones by more than 2 ppm. Much better quantitative agreement is found for the changes of the ^13^C shielding constant between cages. Going from the cage 5^12^6^4^ to the cage 5^12^6^2^ and further the cage 5^12^, the ^13^C shielding constant is changing by -1.50 ppm and -2.40 ppm (experimental values) and -1.49 ppm and -1.27 ppm (calculated results).

Analogous trends are found in this paper for the ethane molecule. The experimental enclathration changes of the ^13^C shielding constant for the ethane molecule amount to -3.05 ppm and -1.35 ppm for cages 5^12^6^2^ and 5^12^6^4^, respectively. The calculated values are -4.54 ppm and -5.66 ppm (and -7.20 ppm for 5^12^ cage). The differences between cages are -1.70 ppm (experiment) and -1.12 ppm (computational) (plus -1.54 ppm when going from 5^12^6^2^ to 5^12^ cage - computational result). Again, as was already seen for the methane, computed changes of the ^13^C shielding constant between cages are quantitatively accurate. Moreover, they are very close (in absolute values) for both ethane and methane molecules enclathrated in 5^12^, 5^12^6^2^ and 5^12^6^4^ cages.

The ^13^C shielding constants obtained for the methane and the ethane show that the size of the cage is the main parameter influencing shielding constant of their carbon atoms. Symmetry of the cage seems to be less important, what is understandable in a connection to the symmetry of the quest molecules. Computed values of the shielding constants’ anisotropies confirm this finding. Thus, the anisotropy of the shielding constant of the carbon nucleus in the cages would also be of interest. Tetrahedral symmetry of the methane molecule together with roughly spherical environment inside 5^12^ and 5^12^6^4^ cages do not favour any particular orientation of guest molecules. Although the 5^12^6^2^ cage provides an oblate cavity, its distortion from sphericity is too small to affect the shielding constant of methane carbon. Values of the static anisotropy (-1.01, 1.00 and 0.76 ppm for the 5^12^, the 5^12^6^2^ and the 5^12^6^4^, respectively) confirm this statement. The experimental anisotropy is expected to be equal zero. On the other hand, the axial symmetry of the ethane results in its orientation in oblate 5^12^6^2^ cage parallel to hexagonal faces. However, the static anisotropy calculated for the ethane molecule in the 5^12^6^2^ cage (6.88 ppm) is much smaller than that found for the CO_2_ (232.96 ppm [43]), which may be attributed to the presence of hydrogen atoms which are lowering axial symmetry of the ethane molecule. Static anisotropy is equal 6.04 and 10.27 ppm for 5^12^ and 5^12^6^4^ cages, respectively. The experimental results, due to averaging, should give the anisotropy equal 0 for 5^12^ and 5^12^6^4^ cages and small value (less than 3 ppm) for the 5^12^6^2^ cage.

The propane, the next molecule studied by us, consists of two methyl and one methylene group. As was already mentioned in the Introduction, the propane molecule is too big to reside inside the 5^12^ cage, therefore we have calculated the NMR properties for bigger cages 5^12^6^2^ and 5^12^6^4^ (Table 1). No experimental data is available for the propane inside the 5^12^6^2^ cage yet, as the sI structure is formed by mixtures containing small amounts of the propane (for example 1.2 mole % in a work of Babu et al. [53]) - too low to give visible signals in experiments published to date.

Let us discuss now the shielding constants calculated for methyl group of the C_3_H_8_ and compare them with these values for the methane and the ethane. The shielding constant values for^13^C for methyl group form growing trend for both experimental and calculated values (for values obtained for the 5^12^, the 5^12^6^2^ and the 5^12^6^4^ with gaseous propane, respectively). The enclathration in the 5^12^6^4^ lowers absolute shielding constant by 1.6 ppm according to experimental data [44], while calculated data give 5.1 ppm for 5^12^6^4^ and 5.9 ppm for 5^12^6^2^. It is analogous to what was previously described for methane and ethane molecules in terms of trend as well as relative changes of the shielding constant values.

The propane ^13^C NMR spectrum showed two peaks corresponding to methyl and methylene carbon nuclei. In this paragraph we would like to discuss the changes of methyl and methylene shielding constants attributed to the studied cages: ${\Delta }\sigma _{(CH_{3}-CH_{2})}$. The calculated values of the ${\Delta }\sigma _{(CH_{3}-CH_{2})}$ for propane-monomer (gas) are equal 3.3 ppm. This value is very close to those presented in [44] for monomer, calculated at the B3LYP/6-311+G(2d,p) level as equal ${\Delta }\sigma _{(CH_{3}-CH_{2})}$ = 3.8 ppm, what means - the methyl carbon nucleus is more shielded than the methylene one in the propane-monomer. In the experimental NMR investigation of the sII structure cage (it means (5^12^6^4^)) by Kida et al. [44], it is the opposite - methyl carbon nucleus is less shielded than methylene one by ${\Delta }\sigma _{(CH_{3}-CH_{2})}$=-0.8 ppm. Our calculations, illustrated in Fig. 2 do not reproduce this result for the methyl-methylene change for 5^12^6^4^ cage (${\Delta }\sigma _{(CH_{3}-CH_{2})}$=+0.7 ppm). However, for the cage 5^12^6^2^ calculated value of the ${\Delta }\sigma _{(CH_{3}-CH_{2})}$ is equal -1.0 ppm. It may be stated that the trend observed experimentally for the 5^12^6^4^ cage encaged the propane molecule [44] is not reproduced in our calculations, but it is assigned correctly to the 5^12^6^2^ cage. Unfortunately, no experimental data exist for the 5^12^6^2^ cage.

Looking for the source of this divergence, let us analyse now changes upon the complexation, i.e. the transfer from the gas phase to clathrate. Experimental ${\Delta }\sigma _{(CH_{3}-CH_{2})}$ in the gas phase amounts to 1.7 ppm. Enclathration by the 5^12^6^4^ cage affects the methyl carbon shielding constant more (-1.6 ppm) and in the opposite direction than shielding constant of methylene carbon (0.9 ppm) and is able to reverse the sequence of their values (in cage 5^12^6^4^ ${\Delta }\sigma _{(CH_{3}-CH_{2})}$ equals -0.8 ppm). Our calculated result for the gas phase give ${\Delta }\sigma _{(CH_{3}-CH_{2})}$ equal to 3.2 ppm. Transfer from the gas phase to 5^12^6^4^ cage changes the methyl carbon shielding constant by -5.1 ppm, namely in the same direction as in the experiment. However, for methylene carbon, the calculated direction of the change of shielding constants value is opposite to that experimentally determined (-2.7 ppm). That is the cause why reversed sequence of shielding constant is not reproduced for the 5^12^6^4^ cage.

#### Hydrogen atoms

The^1^H absolute shielding constants calculated for gaseous guest molecules are all greater by 1 ppm than the corresponding experimental results. These calculated values will be now discussed (see Table 2). The absolute shielding constants for hydrogens of CH_3_ do not depend on the cage enclathrating methane molecule. All are smaller by 0.5 ppm than value characteristic for gaseous molecule. That may be attributed to a lack of steric hindrance in all three cavities for methane molecule. The smaller the cavity, the greater deshielding effect for the ethane ^1^H shielding constants. The changes upon enclathration are -0.80 ppm, -0.68 ppm and -0.49 ppm for the 5^12^, the 5^12^6^2^ and the 5^12^6^4^, respectively. The ^1^H shielding constants for CH_3_ and CH_2_ groups of propane preserve trends observed for ^13^C. Methyl hydrogen’s shielding constants grow with the growing size of the cage towards monomer value. On the other hand, in the CH_2_ group shielding constant for 5^12^6^2^ cage is greater than for 5^12^6^4^ cage. The changes of the absolute values are rather small (-0.28 ppm and -0.45 ppm, respectively), but correlation with trend for methylene carbon shielding constants supports correctness of this ordering.

### Shielding constants of H_2_O.

Shielding constants for oxygen and hydrogen atoms of water molecules forming cages are shown in Table 3. Unfortunately, no experimental data are available for comparison. Computational data presented in Table 3 for 5^12^ and 5^12^6^2^ enclathrating methane are taken from our former publication on the NMR of sI methane hydrate [42], in which a slightly different scheme of cage construction was adopted.

**Table 3:** The comparison of the calculated (B3LYP/aug-cc-pVDZ) ^1^H and the ^17^O shielding constants (in ppm) for the water molecules forming the 5 ^12^, 5 ^12^6^2^ and 5 ^12^6^4^ cages of the CH _4_, C _2_ *H*_6_ and C _3_ *H*_8_ hydrates, divided according to the H-bond patterns and the topological criteria. Values for the monomer water, all in ppm:calculated (this work): *σ*_*O*_ = 325.00; *σ*_*H*_ = 31.34, exp.: *σ*_*O*_ = 322.81 [70]; *σ*_*H*_ = 30.102 [72]

Atom type	CH _4_			C _2_ *H*_6_			C _3_ *H*_8_	
	5 ^12^	5 ^12^6^2^	5 ^12^6^4^	5 ^12^	5 ^12^6^2^	5 ^12^6^4^	5 ^12^6^2^	5 ^12^6^4^
O: (DAA)	289.18 [42]	288.75 [42]	281.94	271.72	278.83	281.59	277.57	280.53
O: (DDA)	288.95 [42]	290.78 [42]	287.10	273.17	280.03	286.00	278.93	285.55
O: 5 ^3^	289.07	289.42	283.96	272.45	279.27	282.29	278.06	281.46
O: 5 ^2^6^1^		289.94	284.00		279.60	284.05	278.44	283.30
H: DAA: d	30.52 [42]	30.51 [42]	30.34	29.63	30.07	30.30	30.10	30.33
H: DAA: H-bond	23.71 [42]	23.73 [42]	23.75	22.39	23.62	23.87	23.61	23.86
H: DDA	26.61 [42]	26.73 [42]	25.84	24.71	25.69	25.91	25.66	25.84
H: 5 ^3^	25.64	25.40	24.95	23.94	24.76	24.76	24.71	24.73
H: 5 ^2^6^1^		25.82	25.17		25.21	25.29	25.22	25.23

#### Oxygen atoms

Let us start analysis of the data in Table 3 from the perspective of H-bond patterns formed by water molecules. Firstly, one can ask the question: what is the change the ^17^O shielding constant with growth of the size of a molecule inside the cavity? The answer one can get looking on the data in Table 3. Although reported values are very close to each other they show a monotonic trend - with growth of the size of molecule residing in cavity the ^17^O shielding constant are getting lower for both DAA and DDA molecules (for example, for 5^12^6^4^ all values of ^17^O shielding constants for DAA water molecules are close to 281.4 ppm, while for DDA water molecules are close to 286.2 ppm)

Secondly, the question may be asked, should there be any change of the^17^O shielding constant between the DDA and DAA types of water molecules. The changes between 5^12^6^2^ and 5^12^6^4^ for DAA type water molecules are equal to 2.76 ppm and 2.96 ppm for cages containing the ethane and the propane, respectively. Analogous changes for DDA type water molecules are equal to 5.97 ppm and 6.62 ppm. Accordingly, the changes for cages enclathrating both guests are quite close and DDA water molecules are more affected by the growth of the size of the cage. Moreover, similar values of ^17^O shielding constants for DAA and DDA groups of water molecules observed for cages independent of the type of guest molecule results also in similar differences between ^17^O shielding values of DAA and DDA type. For 5^12^6^2^ cage those differences are 2.03 ppm, 1.20 ppm and 1.36 ppm for the methane, the ethane and the propane, respectively, while analogous changes for cage 5^12^6^4^ are 5.16 ppm, 4.41 ppm and 5.02 ppm.

Thirdly, we considered a correlation between the ^17^O shielding constants and the strength of H-bonds in clathrate hydrates. The observed monotonic growth of the ^17^O shielding constants may be correlated with decreasing strength of average interaction between water molecules for growing cages. It is analogous to what was previously seen for CO_2_ clathrate cages [43]. It was explained in terms of decreasing strength of H-bonds between water molecules with growing number of hexagonal rings forming cages (i.e. growing size of the cage). However, all water molecules are of DDAA type in real clathrate hydrates and could be involved in formation of two types of cages. Therefore it is hard to draw conclusions on possible differences in the ^17^O shielding constants based on H-bond characteristics presented above.

To continue the answer on above question, much more straightforward for this purpose is analysis based on topological criteria (see our former paper on CO_2_ clathrate hydrates [43]). Crystals of sI and sII hydrates are built of three types of topologically different water molecules denoted 5^6^,5^5^6^1^ and 5^4^6^2^. In single cages those three dimensional topologies are reduced to 5^3^ and 5^2^6^1^ topologies, what was described in detail in [43]. For the 5^12^ cage all water molecules are of 5^3^ topology, while for the 5^12^6^2^ and 5^12^6^4^ cages both 5^3^ and 5^2^6^1^ topologies are present. As to 5^5^6^1^ molecules, half of them is attaining 5^3^ topology, while second half - 5^2^6^1^. This division comes from the fact, that in the crystalline structure the cages of 5^12^ type are neighbouring those of the 5^12^6^2^ type. Therefore, some water molecules forming the pentamers of 5^12^ cages are at the same time forming the hexamers of 5^12^6^2^. However, taking single 5^12^ cage, this information is lost - they are all of the 5^3^ topology. Similar point is true also for the 5^12^6^2^ cage - in CHs some of its water molecules forming only pentamers are located in a junction with neighbouring 5^12^6^2^ cage and form its hexamers, so the 5^3^ topology of single cage is represented by the 5^5^6^1^ in crystal. For sII structure situation is simpler. All 5^6^ of crystal are attaining 5^3^ topology. As for 5^5^6^1^, half of them is reduced to 5^3^ and half to 5^2^6^1^ topologies, analogous as for sI structure. Therefore there is no direct relationship between topologies found in crystal and single cages for any of sI and sII structures. Those differences in H-bond framework and topology between single cages and three dimensional structure should be considered to properly compare our results with experimental ones.

To continue the analysis, the^17^O shielding constant are growing with the cage size for both 5^3^ and 5^2^6^1^ water molecules. The changes between 5^12^6^2^ and 5^12^6^4^ for 5^3^ water molecules are equal to 3.02 ppm and 3.40 ppm for cages containing the ethane and the propane, respectively. Analogous changes for 5^2^6^1^ water molecules are equal to 4.45 ppm and 4.86 ppm. The change in oxygen shielding constants for 5^3^ water molecules going from 5^12^ to 5^12^6^2^ cage enclathrating the methane is 0.35 ppm. It is not possible to obtain such changes for 5^2^6^1^ as all water molecules forming 5^12^ cage are of 5^3^ topology. Generally, a growing trend observed for the ^17^O shielding constants for both types of topologically distinguished water molecules is analogous in the direction and scale observed in H-bond perspective.

The trends described above were observed for single cage, so the question arises - how do they correspond to the three-dimensional lattice of crystalline hydrate? As crystals of hydrates contain water molecules of distinct topologies, analogous to those found in single cages, similar differences between^17^O shielding constants should be expected. In some cases they amount to several ppms, so with increasing sensitivity of the NMR instrumentation and an efficiency of data acquisition they should be observable experimentally in the future.

#### Hydrogen atoms

Oxygen atoms were divided into two categories in the H-bond network perspective, namely those belonging to DAA and DDA water molecules. For hydrogen atoms three categories are needed, as for DAA water molecules one of the hydrogen is involved in H-bond formation and second is not (dangling hydrogen). The values of shielding constants are presented in Table 3.

Generally, for all three categories of protons conclusions are analogous to those drawn for respective^17^O shielding constants. That is: (i) the *σ*(^1^H) is increasing with the growing size of the cage, (ii) the *σ*(^1^H) is almost unaffected by the enclathrated molecule for all cages, (iii) the differences between distinct types of hydrogen atoms are comparable for all cages. The observations made for ^17^O shielding constants in topological perspective also hold for ^1^H values, but respective changes are much smaller and trends less clear.

### Spin-spin coupling constants of guest molecules.

The changes of the SSCC of guest molecules induced by enclathration are caused by distortions of geometry and the interactions with host water molecules. Even for the smallest 5^12^ cage bond lengths are affected only slightly (thousandths of Å). Angles between bonds are more affected, but the overall changes of the SSCC caused by enclathration are small. All ^1^J couplings are dominated by the FC term and variations in its values are mirrored in variations of the SSCC. The PSO and DSO values may make up to 20 % of total SSCC, but as they are opposing in signs they mostly cancel each other out. Changes of their values connected with the enclathration may be appreciable, but again - in opposite directions, so the influence on total SSCC value remain limited. The SD term values are small and almost unaffected by enclathration. Electronic Supplementary Information contain tables summarizing values of SSCC, four distinct terms and interatomic distances.

### Spin-spin coupling constants of H_2_O molecules.

Inter- and intramolecular spin-spin coupling constants for water molecules forming cages may be correlated with strength of interactions those molecules are involved in. For non-polar guest molecules interaction with quest is rather small and dominated by interactions between water molecules, among which H-bonds are by far the strongest. Therefore, it is possible to correlate strength of H-bonds between water molecules forming cages with SSCC characterising those water molecules, as was already done for methane and CO_2_ hydrates [42, 43]. Patterns found for different cages and different guest molecules are analogous. Now, we will briefly describe data obtained for 5^12^6^2^ cage enclathrating ethane molecule. This choice seem to be most representative for all cases, as ethane could be enclathrated in all cages and 5^12^6^2^ cage could contain all three guest molecules described in this paper.

As was already mentioned, DAA-DDA scheme does not provide straightforward connection between single cages and three-dimensional crystal structure. Topological criteria are more reliable for this purpose. However, patterns observed for water SSCC in the H-bond perspective are more explicit than those revealed by topological one. Therefore we will not discuss them in the main body of the article - all data could be found in respective tables in Electronic Supplementary Information.

#### Intramolecular ^1^*J*_*OH*_ and ^2^*J*_*HH*_ coupling constants

**Fig. 3:**
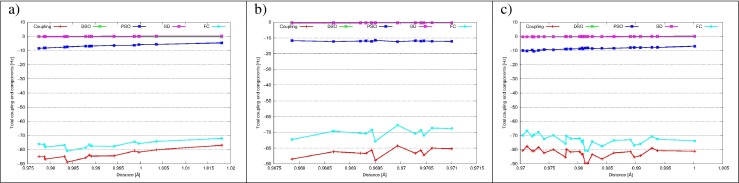
The intramolecular ^1^ *J*_*OH*_ and its components (FC, SD, PSO and DSO) as a function of the intramolecular O-H distance for the cage 5^12^6^2^ of ethane hydrate for: **a** DAA water molecules, hydrogen is H–bond involved; **b** DAA water molecules, dangling hydrogen H^∗^; **c** DDA water molecules

**Fig. 4:**
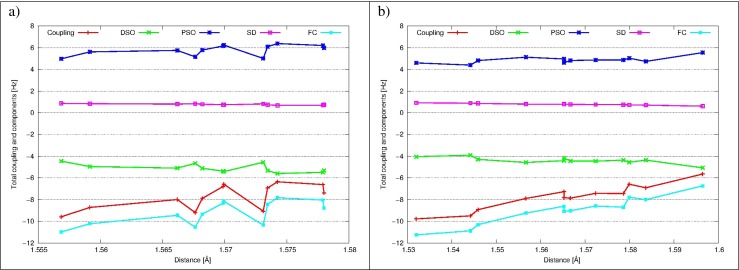
The intramolecular ^2^ *J*_*HH*_ and its components (FC, SD, PSO and DSO) as a function of H ⋯H distance for the cage 5^12^6^2^ of ethane hydrate for: **a** DAA water molecules; **b** DDA water molecules

Intramolecular ^1^ *J*_*OH*_ and ^2^ *J*_*HH*_ coupling constants and their components are presented in Figs. 3 and 4 as a functions of interatomic distance. All ^1^ *J*_*OH*_ couplings are dominated by the FC term, while among others only PSO gives a non-zero contribution. A slight decrease of the absolute value of the shielding is noted for all water H-bond patterns.

Intramolecular ^2^ *J*_*HH*_ coupling constants are also dominated by the FC term. As opposed to ^1^ *J*_*OH*_, also PSO, DSO and SD contributions are non-negligible, but as the sum of their values is close to zero, final coupling is again close to the FC value. For both DAA and DDA water molecules ^2^ *J*_*HH*_ coupling constants are lowering with the interatomic H ⋯H distance (for DDA it falls from -10 Hz to -6 Hz with the elongation of H ⋯H distance from 1.53 Å to 1.60 Å) (more data in Electronic Supplementary Information).

#### Intermolecular ^2*h*^*J*_*OO*_ coupling constants

**Fig. 5:**
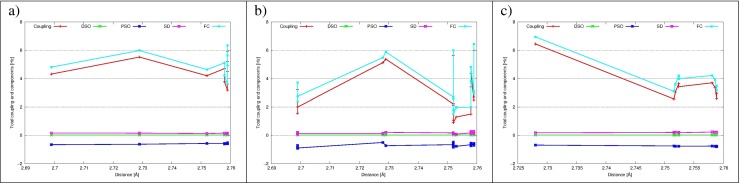
The intermolecular ^2*h*^ *J*_*OO*_ and its components (FC, SD, PSO and DSO) as a function of the O ⋯O distance for the cage 5^12^6^2^ of ethane hydrate for: **a** DAA-DAA water molecules; **b** DDA-DAA water molecules; **c** DDA-DDA water molecules

Intermolecular ^2*h*^ *J*_*OO*_ coupling constants and their components are presented in Fig. 5 as a functions of interatomic distance. Data for DAA-DAA, DAA-DDA and DDA-DDA water pairs are depicted, and their analysis is leading to a conclusion, that final coupling does not depend on the O ⋯O distance in the range 2.270-2.276 Å for none of them. The DSO, PSO and SD terms are low in absolute values and partially cancel each other, so final coupling is determined by the FC term.

#### Intermolecular^1*h*^*J*_*OH*_ coupling constants

**Fig. 6:**
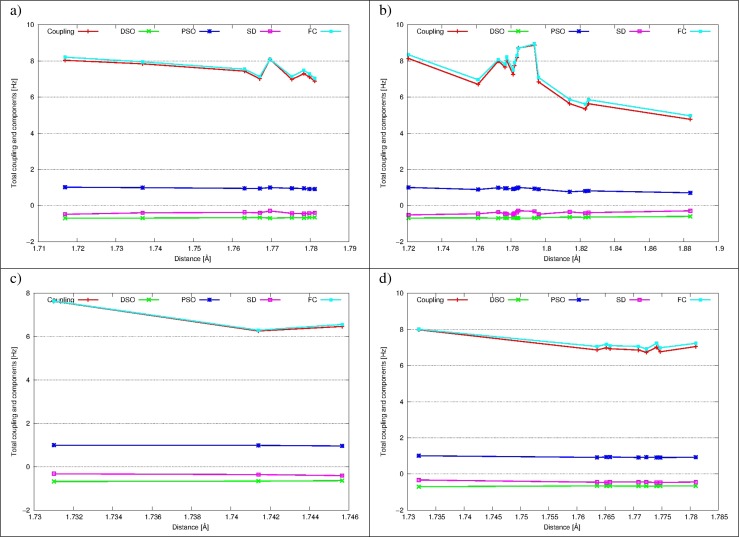
The intermolecular ^1*h*^ *J*_*OH*_ and its components (FC, SD, PSO and DSO) as a function of the O ⋯H distance for the cage 5^12^6^2^ of ethane hydrate for: **a** (O)DAA ⋯(H)DAA H-bonds; **b** (O)DAA ⋯(H)DDA H-bonds; **c** (O)DDA ⋯(H)DAA H-bonds; **d** (O)DDA ⋯(H)DDA H-bonds

Intermolecular^1*h*^ *J*_*OH*_ coupling constants for all four possible pairs of water molecules forming H-bonds, together with their components are presented in the last Fig. 6. Again, final couplings are dominated by the FC term and three other are small and partially cancel each other. A clear descending trend is observed in all cases, but most eminently for (O)DAA ⋯(H)DDA H-bonds, as the range of O ⋯H distances is greatest for this group (from 1.72 Å to 1.89 Å). For all four pairs values of the coupling are similar for respective distances, but as range of those distances is different for every type of H-bond, average ^1*h*^ *J*_*OH*_ coupling constants are also different. In this sense it may be stated, that ^1*h*^ *J*_*OH*_ coupling of (O)DDA-(H)DAA type is weakest, while ^1*h*^ *J*_*OH*_ coupling for three other H-bond types are of comparable strength. This relation was already observed for carbon dioxide clathrate hydrate [43]. Moreover, average values of the ^1*h*^ *J*_*OH*_ and average O ⋯H distances for distinct H-bond types are very close to previously noted, what augments credibility of those conclusions.

## Conclusions

Calculations of NMR parameters (shielding constants and spin-spin coupling constants) for all molecules forming cages 5^12^, 5^12^6^2^ and 5^12^6^4^ of sI and sII clathrate hydrates containing the methane, the ethane and the propane as guest molecules, were performed at DFT/B3LYP/HuzIII-su3 level. Influence of enclathration on guest molecules NMR characteristics was discussed. Two perspectives of the interpretation of the shielding constants and the SSCC of water molecules, based on H-bond characteristics (DDA and DAA water molecules) and topological criteria, were presented. The connection between the calculated and experimental results was described. The most important findings of this paper are:

1. The absolute^13^C shielding constants for the methane, the ethane and the propane inside cages studied are determined. In two cases (ethane in 5^12^ and propane in 5^12^6^2^ cages) neither experimental nor theoretical data existed. Shielding constant values for ^13^C for methyl group form growing trend for both experimental and calculated values (for values obtained for the 5^12^, the 5^12^6^2^ and the 5^12^6^4^ with the gaseous propane, respectively).
2. The change of the methyl/methylene order of the absolute shielding constants is found for the propane enclathrated in 5^12^6^2^ cage. It should be verified experimentally in the near future.
3. The absolute ^1^H shielding constants of the guest molecules for all studied cages are presented for the first time. Analogously to ^13^C shielding constants of propane in 5^12^6^2^ cage, change of the methyl/methylene order of the absolute shielding constants is found also for the ^1^H nuclei.
4. Similar values of the ^17^O shielding constants for the DAA and DDA groups of water molecules observed for cages independent of the type of the guest molecule results also in similar differences between ^17^O shielding values of the DAA and DDA type.
5. The division of water molecules forming host lattice according to topological criteria enables most direct connection between single cages and bulk crystal. Differences of the ^17^O absolute shielding constants may amount to 2 ppm in single cages. It may be expected, that similar differences could be found in real crystals.
  Until now no experimental data exists for some of the structures discussed in this paper, as respective structures were simply not synthesized yet or their spectra could not be obtained to date due to limitations of available experimental NMR techniques. In those cases (the ethane in 5^12^ cage and the propane in 5^12^6^2^ cage) our results are first in the literature presenting ^13^C shielding constants of guest molecules. Additionally, all ^1^H shielding constants and all intramolecular spin-spin coupling constants for the guest molecules were not published yet. Therefore these results should be helpful in an interpretation of future experimental data.

## Electronic supplementary material

Below is the link to the electronic supplementary material.

(PDF 229 KB)
