# Supplementary material for: Calculations of NMR properties for sI and sII clathrate hydrates of methane, ethane and propane
Source: J Mol Model. 2014 Nov 19;20(12):2511. doi: 10.1007/s00894-014-2511-2 (PMC4236610; doi:10.1007/s00894-014-2511-2)
Supplement: Supplementary file 1 — (PDF 229 KB) [file 894_2014_2511_MOESM1_ESM.pdf]

Supplementay Materials for: Calculations of NMR properties for sI and sII clathrate hydrates of methane, ethane and propane

Paweł Siuda and Joanna Sadlej

University of Warsaw, Pasteura 1, 02-093 Warsaw, Poland

1. Methane

Table 1: Shielding constants, anisotropies, dia- and paramagnetic contributions for of ^{17}O and ^{1}H of water molecules forming 5^{12}, $5^{12}6^{2}$ and $5^{12}6^{4}$ cages.

Atom type	σ[ppm]	Anisotropy	Dia. contr.	Para. contr.	Cage	σ[ppm]	Anisotropy	Dia. contr.	Para. contr.	Atom type
O DAA	289.18	27.45	594.66	-305.48	5^{12}	289.07	20.16	584.58	-295.52	5^{3}
	288.75	28.41	614.50	-325.76	$5^{12}6^{2}$	289.42	24.24	585.61	-296.19	
	281.94	22.58	649.43	-367.48	$5^{12}6^{4}$	283.96	25.58	638.42	-354.45	
O DDA	288.95	12.85	574.50	-285.55	5^{12}					$5^{2}6^{1}$
	290.78	10.11	598.28	-307.50	$5^{12}6^{2}$	289.94	15.81	628.53	-338.59	
	287.10	29.47	619.15	-332.05	$5^{12}6^{4}$	284.00	26.91	632.77	-348.78	
H DAA	23.71	21.95	331.81	-308.10	5^{12}	25.64	19.87	335.60	-309.95	5^{3}
	23.73	22.21	331.99	-308.26	$5^{12}6^{2}$	25.40	20.46	353.41	-328.01	
	23.75	21.79	384.78	-361.03	$5^{12}6^{4}$	24.95	21.43	370.89	-345.94	
H DAA*	30.52	12.94	-199.22	229.74	5^{12}					
	30.51	12.60	-228.60	259.11	$5^{12}6^{2}$					
	30.34	12.03	-218.06	248.40	$5^{12}6^{4}$					
H DDA	26.61	18.83	337.49	-310.88	5^{12}					$5^{2}6^{1}$
	26.73	19.07	376.20	-349.47	$5^{12}6^{2}$	25.82	20.02	365.80	-339.98	
	25.84	20.98	406.56	-380.72	$5^{12}6^{4}$	25.17	21.23	403.48	-378.31	

Values for monomer water, all in ppm:calculated (this work):σ_O=325.00; σ_H=31.34
exp.: σ_O=322.81[1]; σ_H=30.102[2]

Table 2: Average values of intramolecular SSCCs of ${}^{X}J_{YZ}$ type and their components (DSO, PSO, SD and FC) for 5^{12}, $5^{12}6^{2}$ and $5^{12}6^{4}$ cages.

Coupled atoms	J	DSO	PSO	SD	FC	Dist.	Cage	J	DSO	PSO	SD	FC	Dist.	Coupled atoms
	-7.65	-5.36	6.04	0.78	-9.11	1.563	5^{12}	-8.21	-4.77	5.36	0.81	-9.62	1.556	
H···H DAA	-7.66	-5.32	5.99	0.78	-9.11	1.562	$5^{12}6^{2}$	-8.19	-4.82	5.42	0.82	-9.62	1.553	5^{3}
	-8.11	-5.22	5.89	0.80	-9.59	1.558	$5^{12}6^{4}$	-7.36	-4.81	5.32	0.74	-8.61	1.561	
	-8.21	-4.77	5.36	0.81	-9.62	1.556	5^{12}							
H···H DDA	-8.04	-4.87	5.48	0.80	-9.45	1.556	$5^{12}6^{2}$	-7.91	-4.92	5.53	0.79	-9.31	1.557	$5^{2}6^{1}$
	-7.86	-4.62	5.10	0.79	-9.13	1.557	$5^{12}6^{4}$	-7.97	-4.63	5.12	0.79	-9.25	1.556	
	-83.59	-0.33	-6.83	0.00	-76.44	0.992	5^{12}	-83.12	-0.32	-8.48	-0.13	-74.19	0.983	
O-H DAA	-83.79	-0.33	-6.88	0.00	-76.57	0.991	$5^{12}6^{2}$	-82.99	-0.32	-8.29	-0.13	-74.25	0.984	5^{3}
	-83.67	-0.34	-6.89	0.05	-76.48	0.991	$5^{12}6^{4}$	-84.86	-0.36	-7.89	-0.09	-76.53	0.981	
	-83.00	-0.25	-12.46	-0.54	-69.75	0.965	5^{12}							
O-H DAA*	-83.41	-0.26	-12.33	-0.52	-70.30	0.965	$5^{12}6^{2}$							
	-82.88	-0.27	-12.21	-0.45	-69.95	0.965	$5^{12}6^{4}$							
	-82.88	-0.32	-9.30	-0.20	-73.06	0.978	5^{12}							
O-H DDA	-83.73	-0.32	-9.34	-0.22	-73.85	0.977	$5^{12}6^{2}$	-84.47	-0.32	-8.56	-0.15	-75.44	0.981	$5^{2}6^{1}$
	-84.69	-0.35	-8.83	-0.19	-75.31	0.977	$5^{12}6^{4}$	-84.24	-0.35	-8.34	-0.12	-75.44	0.981	

Values for monomer (in Hz): calculated (this work): ${}^{1}J_{OH}$=-76.04, ${}^{2}J_{HH}$=-7.72
calculated (CCSD/pz3d2f)[3]: ${}^{1}J_{OH}$=-78.85, ${}^{2}J_{HH}$=-7.84
exp.: ${}^{1}J_{OH}$=-80.6[4], ${}^{2}J_{HH}$=-6.89[2]

Table 3: Coordinates of 5^{12} cage (in Å).

Atom	x	y	z	Atom	x	y	z	Atom	x	y	z
c	-0.002585	0.047505	-0.049200	h1	-0.278712	0.257668	-1.086546	h3	0.721427	-0.772877	-0.021803
				h2	0.431293	0.941084	0.411903	h4	-0.895539	-0.249476	0.507249
o1	2.251893	0.280313	3.275074	h1a	2.703446	0.152688	4.120731	h11a	1.389971	2.932371	1.683041
o2	1.725251	-2.580795	-2.349337	h1	2.856232	-0.064172	2.584695	h11	1.456137	1.937289	2.866516
o3	-0.220204	3.633681	-1.631882	h2a	1.866652	-3.382017	-2.870441	h12a	2.704342	-0.115786	-3.959798
o4	-3.652427	-1.242219	1.000911	h2	1.961378	-2.804215	-1.399663	h12	2.123623	-1.059525	-2.847469
o5	-2.362852	-0.294556	-3.320060	h3a	-0.314201	4.529654	-1.978530	h13a	2.789181	-2.294603	0.567563
o6	-1.683465	2.601158	2.572612	h3	0.608815	3.628227	-1.110161	h13	1.600793	-3.291211	0.748108
o7	0.201943	-3.549555	1.743364	h4a	-3.766550	-0.622145	0.259285	h14a	-0.333966	-2.544209	-2.486992
o8	3.646877	1.141445	-1.089649	h4	-3.189964	-2.026890	0.623655	h14	-1.686948	-1.896380	-2.875220
o9	-0.063846	-1.596754	3.492883	h5a	-1.665764	0.365806	-3.494359	h15a	0.657910	0.969628	-3.363447
o10	3.796182	-0.644335	1.095055	h5	-2.898511	0.076245	-2.593358	h15	-0.161441	2.223464	-2.986763
o11	1.040155	2.772499	2.575917	h6a	-2.026446	3.191243	3.253520	h16a	-1.793935	3.140662	-0.586350
o12	2.341638	-0.108045	-3.065913	h6	-0.697879	2.681190	2.614706	h16	-2.323645	2.834688	0.839442
o13	2.365449	-3.067836	0.161207	h7a	0.094184	-4.384200	2.217145	h17a	2.768675	4.058930	-0.003349
o14	-1.278965	-2.744267	-2.579848	h7	0.080784	-2.823746	2.430925	h17	2.681587	2.578063	-0.469781
o15	-0.106252	1.502222	-3.637944	h8a	4.509275	1.388231	-1.446768	h18a	-2.859771	-0.589710	2.385436
o16	-2.611111	2.957498	-0.087897	h8	3.176317	0.678892	-1.842182	h18	-2.187118	0.708026	2.940220
o17	2.154880	3.321006	-0.099370	h9a	-0.921606	-1.095860	3.388220	h19a	-4.776713	1.022757	-1.299988
o18	-2.335948	-0.231497	3.149104	h9	0.635464	-0.932416	3.381612	h19	-3.437419	1.578194	-0.725475
o19	-3.866423	0.769864	-1.107267	h10a	4.720721	-0.900137	1.198290	h20a	-1.903973	-3.201293	-0.922212
o20	-2.193333	-3.378077	0.000736	h10	3.773270	-0.016041	0.339666	h20	-1.371407	-3.427119	0.522350

Table 4: Coordinates of $5^{12}6^{2}$ cage (in Å).

Atom	x	y	z	Atom	x	y	z	Atom	x	y	z
C	0.262443	-0.204534	-0.100457	H1m	0.863373	0.029680	-0.985398	H3m	0.335151	0.612872	0.625971
				H2m	-0.785167	-0.345092	-0.393452	H4m	0.647384	-1.126676	0.346486
O1	0.373158	-4.270137	-1.091597	H1a	-0.316899	-3.905727	-0.520307	H13a	-1.480233	-3.315581	2.181779
O2	1.902233	4.140085	0.890032	H1	0.379613	-3.674647	-1.880450	H13	-1.100741	-3.424863	3.691562
O3	-2.018680	-4.027973	0.704723	H2a	1.095897	4.372291	0.386339	H14a	-4.755069	-2.960400	-0.883631
O4	-0.489483	4.640911	-0.552970	H2	2.327140	4.975738	1.115867	H14	-4.073206	-1.767561	-0.129917
O5	4.568244	0.318287	0.790533	H3a	-2.008660	-4.964967	0.475054	H15a	-3.146743	4.233126	1.400591
O6	-4.319811	-0.472811	0.834942	H3	-2.732760	-3.603683	0.177443	H15	-3.198890	3.076399	0.344497
O7	4.130405	-1.912706	-1.135545	H4a	-1.259922	4.325357	-0.019670	H16a	-0.393294	3.509412	-2.066883
O8	-4.111941	1.981626	-0.595181	H4	-0.691994	5.554746	-0.787642	H16	-0.102938	3.279423	-3.577192
O9	-2.675627	-0.513937	3.126075	H5a	4.074002	0.204938	1.632944	H17a	-2.675479	0.433237	-2.872049
O10	1.443556	2.259212	3.045054	H5	5.497704	0.167934	1.000364	H17	-1.950707	1.835300	-2.811587
O11	3.731523	2.537761	-0.770850	H6a	-4.231523	0.394902	0.393973	H18a	2.558746	-0.199648	-3.284296
O12	2.977477	0.106050	3.071937	H6	-3.753829	-0.445086	1.633318	H18	3.174780	-1.449825	-2.579672
O13	-1.182878	-2.793869	2.966206	H7a	4.206487	-1.162433	-0.525742	H19a	2.439966	-3.402652	1.186592
O14	-3.892014	-2.564505	-0.712302	H7	3.682439	-2.616876	-0.609941	H19	1.960636	-4.032324	-0.149898
O15	-2.585928	3.634295	0.893503	H8a	-3.602466	1.751685	-1.446083	H20a	-0.380530	-1.814285	-2.856714
O16	-0.441304	2.846411	-2.783549	H8	-4.983843	2.271658	-0.890242	H20	1.115862	-1.986937	-3.269706
O17	-2.821888	1.394473	-2.745122	H9a	-2.129317	-1.338593	3.122537	H21a	-1.714638	1.130102	3.031743
O18	2.586652	-1.175730	-3.321810	H9	-3.167931	-0.533257	3.955417	H21	-1.727895	2.479028	2.265416
O19	2.765942	-3.804291	0.360666	H10a	0.466149	2.164082	3.074349	H22a	-2.443407	-1.923067	-3.646969
O20	0.249114	-2.474516	-3.178334	H10	1.621980	2.902063	2.335931	H22	-2.994241	-1.862094	-2.189140
O21	-1.327026	2.025806	3.025834	H11a	3.146944	3.037499	-0.176308	H23a	2.876470	1.977431	-2.271207
O22	-2.515766	-1.349866	-2.872413	H11	4.053563	1.783681	-0.238442	H23	1.420318	1.840329	-2.760468
O23	2.333975	1.674025	-3.032387	H12a	2.397257	0.920050	3.062793	H24a	2.116984	-1.580157	2.831121
O24	1.765277	-2.487242	2.765293	H12	3.407197	0.106593	3.935229	H24	0.799947	-2.396787	2.756532

5

Table 5: Coordinates of $5^{12}6^4$ cage (in Å).

Atom	x	y	z	Atom	x	y	z	Atom	x	y	z
c	0.124045	0.125468	-0.181052	h2	-0.743857	0.273991	0.355761	h4	0.476057	-1.031762	0.325773
h1	-0.156899	-0.368541	-1.212458	h3	0.923137	0.626390	-0.191089				
o1	0.475227	-4.124567	1.871961	h1a	1.388494	-3.807080	1.706082	h15a	4.016155	1.539319	-0.046975
o2	2.981497	-3.172023	1.303666	h1b	0.075507	-3.487630	2.485388	h15b	3.579996	2.376565	-1.281168
o3	-0.872763	-4.545687	-0.488709	h2a	3.175183	-2.344763	1.776647	h16a	-4.480439	-0.731100	0.431707
o4	3.260385	-2.974826	-1.425897	h2b	3.048321	-2.976619	0.353385	h16b	-4.540363	-2.308049	0.379191
o5	-0.651109	-2.553071	3.828728	h3a	-0.220606	-4.272778	-1.156173	h17a	-1.840545	-1.734097	-3.613715
o6	3.482038	-0.982208	2.891539	h3b	-0.430031	-4.417664	0.381082	h17b	-2.894820	-2.255233	-2.555362
o7	0.888565	-3.854789	-2.461938	h4a	3.444500	-2.095745	-1.840716	h18a	2.218192	-0.078749	-3.520693
o8	1.242314	-0.674650	4.428515	h4b	4.061163	-3.497793	-1.554619	h18b	1.238338	0.967349	-4.131286
o9	-3.165280	-3.123880	-1.027217	h5a	-1.505252	-2.094909	3.655289	h19a	-1.481304	1.697777	3.836297
o10	3.693985	-0.516916	-2.582550	h5b	-0.842131	-3.237841	4.480342	h19b	-2.473755	0.587533	3.386624
o11	3.915637	1.475701	1.734885	h6a	3.688877	-0.114080	2.481026	h20a	1.551394	3.740839	1.834395
o12	-2.943628	-1.131260	3.290219	h6b	4.226900	-1.195375	3.466550	h20b	1.880886	3.700512	3.373404
o13	-0.196259	-2.024678	-4.178653	h7a	1.772022	-3.610900	-2.109249	h21a	-4.260266	0.956452	-1.142035
o14	0.235851	1.859921	4.238169	h7b	1.021163	-4.652443	-2.988671	h21b	-5.486230	1.220033	-0.220937
o15	4.194526	1.672899	-0.994678	h8a	2.011543	-0.848912	3.859764	h22a	1.885816	3.972387	-1.030997
o16	-4.291617	-1.552380	0.929550	h8b	0.594396	-1.379573	4.241588	h22b	2.683403	4.286462	-2.335387
o17	-2.755061	-1.595181	-3.264736	h9a	-2.431415	-3.777268	-0.879778	h23a	-3.250575	0.041515	-2.938563
o18	1.597933	0.059238	-4.251773	h9b	-2.882017	-2.388330	-0.466886	h23b	-4.223938	1.127630	-3.492746
o19	-2.405789	1.544702	3.538497	h10a	3.913555	0.252086	-1.989805	h24a	1.237144	2.860062	-2.952183
o20	1.947205	3.199119	2.551459	h10b	4.302958	-0.454091	-3.328413	h24b	-0.205217	2.747569	-3.499400
o21	-4.567792	0.922408	-0.198456	h11a	3.229078	2.099449	2.075260	h25a	-3.774843	2.118032	0.828342
o22	2.379414	3.562793	-1.773729	h11b	4.760257	1.882625	1.967421	h25b	-3.023457	2.451999	2.155436
o23	-3.615115	0.968630	-2.760394	h12a	-3.644085	-1.132353	3.955766	h26a	-0.013736	4.361887	0.140344
o24	0.737881	2.623049	-3.747433	h12b	-3.396378	-1.353139	2.438009	h26b	1.020206	5.473060	0.454687
o25	-3.404656	2.860610	1.338992	h13a	0.407173	-1.251904	-4.254679	h27a	-1.776595	3.362186	-1.913743
o26	0.948338	4.515029	0.351953	h13b	0.232556	-2.630731	-3.553104	h27b	-2.399383	2.267270	-2.822797
o27	-1.820921	3.052543	-2.833515	h14a	0.670627	2.100175	3.407681	h28a	-2.279957	3.701779	0.304097
o28	-1.693303	4.199807	-0.347720	h14b	0.570516	0.947080	4.411903	h28b	-2.171705	5.019585	-0.525174

2. Ethane

Table 6: Shielding constants of ^{13}C and ^{1}H, anisotropies, dia- and paramagnetic contributions for ethane in 5^{12}, $5^{12}6^{2}$ and $5^{12}6^{4}$ cages and monomer ethane.

Atom	Molecule	σ[ppm]	Anisotropy	Diamagnetic cont.	Paramagnetic cont.
**C**H_3	monomer	171.68	10.44	322.90	-151.22
	5^{12}	164.06	5.23	803.15	-639.09
		164.89	6.85	803.20	-638.30
	$5^{12}6^{2}$	163.98	5.83	843.94	-679.95
		168.05	7.94	850.51	-682.46
	$5^{12}6^{4}$	168.22	10.02	885.62	-717.40
		166.07	10.52	882.98	-716.91
C**H**$_3$	monomer	30.92	5.91	26.93	3.99
	5^{12}	29.88	6.13	505.41	-475.52
		30.36	6.16	506.48	-476.13
	$5^{12}6^{2}$	30.04	5.68	544.39	-514.35
		30.43	6.26	557.25	-526.82
	$5^{12}6^{4}$	30.28	5.88	590.31	-560.03
		30.57	5.58	586.73	-556.16

Table 7: Shielding constants, anisotropies, dia- and paramagnetic contributions for of ^{17}O and ^{1}H of water molecules forming 5^{12}, $5^{12}6^{2}$ and $5^{12}6^{4}$ cages.

Atom type	σ[ppm]	Anisotropy	Dia. contr.	Para. contr.	Cage	σ[ppm]	Anisotropy	Dia. contr.	Para. contr.	Atom type
	271.72	25.63	608.39	-336.68	5^{12}	272.44	21.82	589.19	-316.75	
O DAA	278.83	22.26	627.71	-348.88	$5^{12}6^{2}$	279.27	26.44	591.07	-311.80	5^{3}
	281.59	22.49	648.04	-366.45	$5^{12}6^{4}$	282.29	25.28	638.84	-356.55	
	273.17	29.43	569.99	-296.82	5^{12}					
O DDA	280.03	33.25	596.23	-316.20	$5^{12}6^{2}$	279.60	29.07	632.87	-353.27	$5^{2}6^{1}$
	286.00	30.46	619.67	-333.67	$5^{12}6^{4}$	284.05	26.67	633.03	-348.98	
	22.39	20.53	348.63	-326.23	5^{12}	23.94	19.96	336.48	-312.54	
H DAA	23.62	21.00	356.19	-332.57	$5^{12}6^{2}$	24.76	20.45	369.36	-344.60	5^{3}
	23.87	21.48	378.55	-354.69	$5^{12}6^{4}$	24.76	21.25	377.14	-352.37	
	29.63	12.50	-178.06	207.68	5^{12}					
H DAA*	30.07	12.29	-192.52	222.59	$5^{12}6^{2}$					
	30.30	12.15	-213.76	244.05	$5^{12}6^{4}$					
	24.71	19.68	330.41	-305.70	5^{12}					
H DDA	25.69	20.00	387.29	-361.61	$5^{12}6^{2}$	25.21	20.23	383.70	-358.49	$5^{2}6^{1}$
	25.91	20.60	413.82	-387.91	$5^{12}6^{4}$	25.29	20.85	405.44	-380.14	

Values for monomer water, all in ppm:calculated (this work):σ_O=325.00; σ_H=31.34
exp.: σ_O=322.81[1]; σ_H=30.102[2]

Table 8: SSCCs and their components (DSO, PSO, SD and FC) for ethane molecules enclathrated by 5^{12}, $5^{12}6^2$ and $5^{12}6^4$ cages and for single ethane molecule.

Coupled atoms	Molecule	J[Hz]	DSO[Hz]	PSO[Hz]	SD[Hz]	FC[Hz]	Distance[Å]
$\mathbf{C}H_3$-$\mathbf{C}H_3$	monomer	34.47	0.11	0.02	1.10	33.24	1.529
	5^{12}	32.83	0.24	-0.12	1.11	31.60	1.522
	$5^{12}6^2$	33.70	0.22	-0.15	1.08	32.54	1.532
	$5^{12}6^4$	34.39	0.20	-0.13	1.09	33.23	1.531
$\mathbf{CH}_3$	monomer	131.95	0.50	1.35	-0.05	130.16	1.091
	5^{12}	136.41	1.12	0.62	-0.07	134.73	1.093
		130.85	1.14	0.79	-0.07	128.99	1.095
	$5^{12}6^2$	135.37	1.04	0.73	-0.05	133.66	1.094
		131.32	1.00	0.89	-0.06	129.49	1.097
	$5^{12}6^4$	135.01	0.88	0.89	-0.07	133.30	1.098
		131.82	0.91	0.97	-0.06	130.01	1.099
$C\mathbf{H}_3$	monomer	-13.95	-2.90	3.05	0.41	-14.52	1.760
	5^{12}	-14.78	-0.53	0.78	0.42	-15.45	1.763
		-12.87	-0.52	0.81	0.40	-13.56	1.771
	$5^{12}6^2$	-14.46	-0.76	1.01	0.42	-15.12	1.765
		-13.08	-0.93	1.19	0.40	-13.74	1.773
	$5^{12}6^4$	-14.43	-1.29	1.50	0.42	-15.05	1.771
		-13.40	-1.20	1.44	0.41	-14.05	1.773

Table 9: Average values of intramolecular SSCCs of ${}^{X}J_{YZ}$ type and their components (DSO, PSO, SD and FC) for 5^{12}, $5^{12}6^{2}$ and $5^{12}6^{4}$ cages.

Coupled atoms	J	DSO	PSO	SD	FC	Dist.	Cage	J	DSO	PSO	SD	FC	Dist.	Coupled atoms
	-7.84	-5.30	5.99	0.80	-9.32	1.559	5^{12}	-8.27	-4.73	5.34	0.82	-9.70	1.553	
H···H DAA	-7.76	-5.11	5.78	0.77	-9.20	1.570	$5^{12}6^{2}$	-8.13	-4.27	4.74	0.81	-9.41	1.560	5^{3}
	-7.81	-5.24	5.93	0.79	-9.29	1.561	$5^{12}6^{4}$	-7.68*	-4.62*	5.08*	0.76*	-8.91*	1.561*	
	-8.27	-4.73	5.34	0.82	-9.70	1.553	5^{12}							
H···H DDA	-7.75	-4.38	4.87	0.78	-9.02	1.566	$5^{12}6^{2}$	-7.48	-4.46	4.96	0.76	-8.73	1.569	$5^{2}6^{1}$
	-7.79	-4.59	5.08	0.78	-9.07	1.559	$5^{12}6^{4}$	-7.80	-4.58	5.08	0.79	-9.08	1.559	
	-84.00	-0.38	-6.29	0.14	-77.47	1.014	5^{12}	-84.41	-0.38	-7.63	-0.02	-76.38	1.001	
O-H DAA	-82.25	-0.36	-6.91	0.07	-75.06	0.996	$5^{12}6^{2}$	-81.39	-0.36	-7.98	-0.05	-72.99	0.989	5^{3}
	-83.97	-0.35	-6.98	0.03	-76.67	0.990	$5^{12}6^{4}$	-84.35	-0.36	-7.77	-0.07	-76.15	0.983	
	-84.76	-0.28	-11.84	-0.44	-72.19	0.976	5^{12}							
O-H DAA*	-82.82	-0.27	-12.00	-0.41	-70.13	0.970	$5^{12}6^{2}$							
	-83.30	-0.28	-12.15	-0.45	-70.43	0.966	$5^{12}6^{4}$							
	-84.62	-0.38	-8.30	-0.10	-75.84	0.994	5^{12}							
O-H DDA	-82.49	-0.37	-8.78	-0.16	-73.18	0.982	$5^{12}6^{2}$	-83.33	-0.37	-8.32	-0.11	-74.53	0.985	$5^{2}6^{1}$
	-84.25	-0.36	-8.87	-0.20	-74.82	0.977	$5^{12}6^{4}$	-84.13	-0.36	-8.30	-0.13	-75.35	0.981	

Values for monomer (in Hz): calculated (this work): ${}^{1}J_{OH}$=-76.04, ${}^{2}J_{HH}$=-7.72
calculated (CCSD/pz3d2f)[3]: ${}^{1}J_{OH}$=-78.85, ${}^{2}J_{HH}$=-7.84
exp.: ${}^{1}J_{OH}$=-80.6[4], ${}^{2}J_{HH}$=-6.89[2]

Table 10: Average values of intermolecular SSCCs of ${}^{2h}J_{OO}$ type and their components (DSO, PSO, SD and FC) for 5^{12}, $5^{12}6^2$ and $5^{12}6^4$ cages. Brackets contain standard deviation in % of average value.

Coupled atoms	Cage	J[Hz]	DSO[Hz]	PSO[Hz]	SD[Hz]	FC[Hz]	$r_{C \cdots O}$[Å]
DAA-DAA	5^{12}	4.80 (30)	0.02 (3)	-0.62 (3)	0.17 (11)	5.24 (28)	2.759 (0)
	$5^{12}6^2$	4.60 (18)	0.02 (4)	-0.59 (6)	0.14 (11)	5.03 (16)	2.748 (1)
	$5^{12}6^4$	4.99 (17)	0.02 (3)	-0.58 (10)	0.14 (18)	5.41 (15)	2.742 (0)
DAA-DDA	5^{12}	3.09 (47)	0.02 (5)	-0.68 (9)	0.19 (18)	3.55 (39)	2.752 (0)
	$5^{12}6^2$	2.96 (55)	0.02 (8)	-0.69 (17)	0.16 (32)	3.47 (44)	2.743 (1)
	$5^{12}6^4$	2.84 (52)	0.01 (5)	-0.70 (12)	0.15 (30)	3.37 (42)	2.745 (0)
DDA-DDA	5^{12}	4.32 (17)	0.02 (4)	-0.79 (11)	0.22 (8)	4.87 (15)	2.749 (1)
	$5^{12}6^2$	3.53 (31)	0.01 (6)	-0.77 (4)	0.21 (9)	4.07 (27)	2.752 (0)
	$5^{12}6^4$	4.07 (21)	0.01 (3)	-0.77 (4)	0.18 (6)	4.64 (19)	2.747 (0)

Table 11: Average values of intermolecular SSCCs of ${}^{1h}J_{OH}$ type and their components (DSO, PSO, SD and FC) for 5^{12}, $5^{12}6^2$ and $5^{12}6^4$ cages. Brackets contain standard deviation in % of average value.

Coupled atoms	Cage	J[Hz]	DSO[Hz]	PSO[Hz]	SD[Hz]	FC[Hz]	$r_{O\cdots H}$[Å]
	5^{12}	7.26 (3)	-0.68 (1)	0.98 (2)	-0.42 (12)	7.38 (3)	1.754 (1)
(O)DAA···(H)DAA	$5^{12}6^2$	7.41 (6)	-0.68 (2)	0.96 (3)	-0.41 (12)	7.54 (6)	1.763 (1)
	$5^{12}6^4$	7.33 (6)	-0.68 (1)	0.98 (2)	-0.40 (16)	7.44 (5)	1.761 (1)
	5^{12}	7.74 (8)	-0.68 (2)	0.96 (4)	-0.46 (8)	7.91 (7)	1.769 (1)
(O)DAA···(H)DDA	$5^{12}6^2$	7.17 (17)	-0.67 (4)	0.90 (10)	-0.41 (17)	7.35 (16)	1.792 (2)
	$5^{12}6^4$	6.85 (17)	-0.66 (4)	0.87 (11)	-0.41 (16)	7.05 (17)	1.797 (2)
	5^{12}	6.51 (4)	-0.65 (1)	1.00 (2)	-0.41 (4)	6.57 (4)	1.732 (1)
(O)DDA···(H)DAA	$5^{12}6^2$	6.79 (9)	-0.66 (2)	0.98 (2)	-0.36 (9)	6.83 (8)	1.739 (0)
	$5^{12}6^4$	6.33 (12)	-0.66 (1)	0.97 (1)	-0.37 (9)	6.38 (12)	1.747 (1)
	5^{12}	7.53 (6)	-0.65 (1)	0.95 (2)	-0.42 (8)	7.65 (6)	1.757 (1)
(O)DDA···(H)DDA	$5^{12}6^2$	7.02 (5)	-0.67 (2)	0.93 (3)	-0.44 (9)	7.19 (4)	1.767 (1)
	$5^{12}6^4$	7.25 (4)	-0.67 (1)	0.92 (2)	-0.43 (5)	7.43 (4)	1.765 (0)

Table 12: Coordinates of 5^{12} cage (in Å).

Atom	x	y	z	Atom	x	y	z	Atom	x	y	z
c1	0.679876	0.084233	0.339877	h1c1	0.977203	-0.804823	0.900323	h1c2	-1.430214	-0.411488	0.297394
c2	-0.628380	-0.150198	-0.403054	h2c1	0.610988	0.907788	1.058337	h2c2	-0.924298	0.729948	-0.983053
				h3c1	1.485902	0.310825	-0.362269	h3c2	-0.525394	-0.975041	-1.112547
o1	-2.473865	-0.340582	-2.797027	h1a	-3.089103	-0.534046	-3.531309	h11a	-1.145177	2.569993	-2.387454
o2	-1.528302	-1.207607	3.223887	h1b	-2.946339	-0.402840	-1.917929	h11b	-1.568560	1.150168	-3.071535
o3	1.067735	3.582980	0.358911	h2a	-1.562969	-1.744537	4.037980	h12a	-2.000432	1.906496	3.994094
o4	3.058872	-2.130456	-0.776538	h2b	-2.064744	-1.649048	2.468700	h12b	-1.568543	0.531919	3.216860
o5	2.536086	0.292749	2.801643	h3a	1.438988	4.485155	0.381097	h13a	-3.299498	-1.628219	0.524380
o6	1.590522	1.159775	-3.219270	h3b	0.217610	3.559500	-0.165617	h13b	-2.262090	-2.874683	0.620912
o7	-1.005516	-3.630813	-0.354294	h4a	3.365358	-1.320303	-0.304274	h14a	0.204023	-1.665745	3.034323
o8	-2.996653	2.082624	0.781154	h4b	2.541884	-2.726436	-0.157756	h14b	1.749260	-1.259595	3.139345
o9	-0.802653	-2.534534	-2.877286	h5a	2.011989	1.127825	2.911857	h15a	-0.051858	2.140260	2.969558
o10	-3.850759	-0.255326	-0.407526	h5b	3.049161	0.276993	1.955528	h15b	0.990460	2.891303	1.989308
o11	-1.041589	1.986154	-3.175640	h6a	1.994119	1.285342	-4.099268	h16a	2.309725	2.685261	-0.536977
o12	-1.682852	1.545336	3.147178	h6b	0.623014	1.470366	-3.234337	h16b	2.552440	1.868909	-1.917151
o13	-2.930095	-2.318607	1.123059	h7a	-1.017848	-4.605743	-0.409338	h17a	-1.780025	4.361537	-1.153008
o14	1.102875	-2.034331	3.180353	h7b	-0.909709	-3.240266	-1.299651	h17b	-1.965607	2.956763	-0.336776
o15	0.865211	2.485945	2.882463	h8a	-3.770486	2.623273	1.038819	h18a	2.286580	-1.892638	-2.339845
o16	2.992315	2.270774	-1.118443	h8b	-2.459778	1.869538	1.622508	h18b	1.683574	-0.609655	-3.117160
o17	-1.369337	3.505320	-0.932392	h9a	0.173351	-2.286114	-3.044029	h19a	4.881916	0.164102	0.509348
o18	1.745409	-1.593924	-3.142000	h9b	-1.351964	-1.716465	-2.918796	h19b	3.645645	0.986541	-0.184859
o19	3.912979	0.207494	0.412143	h10a	-4.825388	-0.265961	-0.449008	h20a	1.407570	-3.101523	1.831477
o20	1.431556	-3.553152	0.937009	h10b	-3.510654	0.583417	0.022936	h20b	0.545432	-3.490816	0.508842

Table 13: Coordinates of $5^{12}6^{2}$ cage (in Å).

Atom	x	y	z	Atom	x	y	z	Atom	x	y	z
c1	-0.500418	-0.954308	-0.044759	h1c1	-0.901252	-1.335435	-0.984866	h1c2	0.411822	0.827437	-0.917924
c2	0.698295	-0.035738	-0.300923	h2c1	-1.308081	-0.435197	0.490628	h2c2	1.135271	0.339869	0.634082
				h3c1	-0.218922	-1.827226	0.549492	h3c2	1.479300	-0.582915	-0.834693
o1	-3.827491	-1.985867	-0.889751	h1	-3.684337	-1.203160	-0.332904	h13	-3.336728	-0.003859	2.179092
o2	4.296572	-0.156080	0.826893	h1a	-3.314831	-1.783304	-1.715420	h13a	-3.170268	-0.377007	3.693141
o3	-4.343628	0.111858	0.728384	h2	4.175941	0.631917	0.247459	h14	-4.569536	3.088947	-0.786895
o4	3.818963	2.039090	-0.669210	h2a	5.249392	-0.258236	0.968474	h14a	-3.162252	2.908111	-0.120306
o5	1.990731	-3.845387	0.682926	h3	-5.245214	-0.231031	0.630939	h15	2.769210	4.652281	1.211071
o6	-2.037787	3.801165	0.872350	h3a	-4.288642	0.949336	0.204897	h15a	1.593228	4.099650	0.315660
o7	-0.137466	-4.291125	-0.916232	h4	3.307230	2.678241	-0.103512	h16	2.924824	1.568993	-2.132877
o8	0.128938	4.344348	-0.642730	h4a	4.626414	2.516442	-0.918719	h16a	2.941717	1.251100	-3.670206
o9	-1.232019	2.256013	3.011202	h5	1.723592	-3.405115	1.528228	h17	-0.798993	2.247897	-2.967304
o10	2.577587	-0.123950	2.983863	h5a	2.026075	-4.797495	0.860715	h17a	0.787757	2.072050	-2.959884
o11	3.850114	-2.383462	-0.737168	h6	-1.224559	3.963143	0.348717	h18	0.917667	-2.201751	-3.034718
o12	1.132000	-2.436863	2.893607	h6a	-1.761060	3.245842	1.634086	h18a	-0.067750	-3.218459	-2.325300
o13	-2.677606	-0.056900	2.920946	h7	0.562635	-3.935413	-0.343259	h19	-2.199670	-3.426198	1.400083
o14	-3.860691	2.433684	-0.681833	h7a	-0.983453	-4.170641	-0.409008	h19a	-2.965559	-3.233996	0.031619
o15	2.386090	3.833085	0.859040	h8	0.112832	3.706565	-1.437699	h20	-2.080649	-0.173275	-2.788858
o16	2.369326	1.274851	-2.888289	h8a	-0.017254	5.220982	-1.032366	h20a	-1.465073	-1.594352	-3.094885
o17	0.055746	2.720207	-2.871686	h9	-1.714431	1.385404	3.015343	h21	0.549546	2.273346	2.980409
o18	-0.009616	-2.531414	-3.036820	h9a	-1.445737	2.678535	3.857134	h21a	1.772023	2.889166	2.239072
o19	-2.430592	-3.873521	0.558308	h10	2.192341	0.785986	3.039633	h22	-3.038945	1.493582	-3.711060
o20	-2.323196	-1.086058	-3.020217	h10a	3.224721	-0.099965	2.251172	h22a	-3.078564	1.926409	-2.198001
o21	1.515558	2.414455	3.050151	h11	3.893974	-1.624322	-0.125725	h23	3.036479	-1.828936	-2.202226
o22	-2.483614	1.660975	-2.936246	h11a	3.235460	-3.011659	-0.303010	h23a	2.338867	-0.543316	-2.759468
o23	2.529427	-1.470806	-2.972221	h12	1.578012	-1.531748	2.859962	h24	-0.637783	-2.590929	2.835222
o24	-1.615577	-2.595305	2.854657	h12a	1.401629	-2.793919	3.754182	h24a	-1.849808	-1.666026	2.685250

Table 14: Coordinates of $5^{12}6^4$ cage (in Å).

Atom	x	y	z	Atom	x	y	z	Atom	x	y	z
c1	0.345070	-0.430905	0.436524	h1c1	0.039247	-0.458121	1.491180	h1c2	-0.562991	1.504148	-0.022162
c2	-0.576384	0.467964	-0.391507	h2c1	1.385590	-0.078416	0.400897	h2c2	-0.285242	0.484520	-1.449901
				h3c1	0.334593	-1.460651	0.061177	h3c2	-1.616284	0.113583	-0.345584
o1	2.413885	-3.393788	1.886441	h1a	3.063863	-2.693229	1.671756	h15a	2.874252	3.163478	-0.305536
o2	4.179412	-1.425883	1.163976	h1b	1.802421	-3.010464	2.538547	h15b	2.058967	3.681811	-1.522249
o3	1.301829	-4.469928	-0.387856	h2a	4.004976	-0.585834	1.621092	h16a	-3.603382	-2.650747	0.739590
o4	4.213399	-1.224611	-1.579291	h2b	4.064005	-1.259023	0.213897	h16b	-3.536138	-4.138257	1.214352
o5	0.783932	-2.436620	3.885007	h3a	1.720998	-3.947580	-1.091263	h17a	-0.981489	-2.522930	-3.478009
o6	3.695503	0.808696	2.693573	h3b	1.681812	-4.126788	0.453966	h17b	-1.636879	-3.353896	-2.292874
o7	2.461463	-3.126176	-2.466998	h4a	3.980897	-0.373408	-2.024327	h18a	1.856280	0.789646	-3.631301
o8	1.634921	0.118902	4.352319	h4b	5.166057	-1.332725	-1.694316	h18b	0.491785	1.258259	-4.220463
o9	-1.408783	-4.269645	-0.811706	h5a	-0.196423	-2.394215	3.699950	h19a	-1.878114	0.937481	3.911312
o10	3.423184	1.116198	-2.788978	h5b	0.879205	-3.073108	4.604241	h19b	-2.909876	0.718709	2.767026
o11	2.905288	3.149505	1.483883	h6a	3.486618	1.672090	2.276489	h20a	-0.220487	4.087816	1.686578
o12	-1.926678	-2.236337	3.461157	h6b	4.431860	0.965161	3.297521	h20b	0.179652	4.296577	3.196893
o13	0.583796	-2.056234	-4.141167	h7a	3.161812	-2.507488	-2.166462	h21a	-4.198939	-1.141548	-0.907445
o14	-0.425840	1.907688	4.188756	h7b	2.908921	-3.812257	-2.977415	h21b	-5.390932	-1.554521	-0.005842
o15	2.939277	3.350777	-1.259383	h8a	2.340387	0.277239	3.702106	h22a	-0.156457	4.353355	-1.189528
o16	-3.038734	-3.312478	1.186859	h8b	1.353693	-0.811456	4.228497	h22b	0.340206	4.930003	-2.554434
o17	-1.844186	-2.806023	-3.087650	h9a	-0.443743	-4.388997	-0.626989	h23a	-3.004406	-1.566274	-2.757388
o18	1.222255	0.611916	-4.342458	h9b	-1.775233	-3.840147	-0.023483	h23b	-4.426095	-1.115434	-3.228481
o19	-2.660264	0.397977	3.645346	h10a	3.291954	1.922993	-2.220850	h24a	-0.323651	2.970204	-3.045625
o20	0.406176	3.815916	2.390538	h10b	3.874270	1.423883	-3.587922	h24b	-1.577333	2.206533	-3.540919
o21	-4.465902	-1.278933	0.046185	h11a	2.026644	3.403321	1.855438	h25a	-4.360633	0.173768	1.017843
o22	0.428015	4.175960	-1.956439	h11b	3.473569	3.917741	1.631043	h25b	-4.891715	1.048511	2.209864
o23	-3.756768	-0.898785	-2.566451	h12a	-2.229232	-1.303575	3.526859	h26a	-1.978914	3.878072	0.081550
o24	-0.690327	2.519154	-3.821260	h12b	-2.324860	-2.619880	2.660917	h26b	-1.541126	5.342701	0.325265
o25	-4.248508	1.031830	1.490610	h13a	0.767444	-1.098620	-4.260622	h27a	-3.038507	1.996576	-1.828642
o26	-1.182067	4.449769	0.235802	h13b	1.269193	-2.382240	-3.533432	h27b	-3.278344	0.805221	-2.781227
o27	-3.118308	1.769366	-2.767742	h14a	-0.175935	2.304262	3.342753	h28a	-3.766846	2.324825	0.382087
o28	-3.416490	2.940057	-0.307609	h14b	0.299000	1.249929	4.347933	h28b	-4.187921	3.350861	-0.717716

3. Propane

Table 15: Shielding constants of ^{13}C and ^{1}H, anisotropies, dia- and paramagnetic contributions for propane in $5^{12}6^{2}$ and $5^{12}6^{4}$ cages and monomer propane.

Atom	Molecule	σ[ppm]	Anisotropy	Diamagnetic cont.	Paramagnetic cont.
$\mathbf{C}H_3$	monomer	162.18	20.67	328.81	-166.64
	$5^{12}6^{2}$	157.07	16.37	857.53	-700.46
		155.55	17.23	853.07	-697.52
	$5^{12}6^{4}$	156.60	19.66	891.21	-734.60
		157.53	18.59	893.55	-736.02
$\mathbf{C}H_2$	monomer	158.96	5.26	366.49	-207.53
	$5^{12}6^{2}$	157.32	5.32	886.52	-729.20
	$5^{12}6^{4}$	156.23	4.77	922.56	-766.33
$C\mathbf{H}_3$	monomer	30.94	5.67	24.63	6.31
	$5^{12}6^{2}$	29.86	6.63	554.20	-524.35
		30.48	5.40	547.32	-516.84
	$5^{12}6^{4}$	30.07	5.64	589.48	-559.41
		30.50	5.03	586.40	-555.90
$C\mathbf{H}_2$	monomer	30.48	5.55	43.46	-12.98
	$5^{12}6^{2}$	30.20	5.77	560.78	-530.58
	$5^{12}6^{4}$	30.03	5.55	595.67	-565.64

Table 16: Shielding constants, anisotropies, dia- and paramagnetic contributions for of ^{17}O and ^{1}H of water molecules forming $5^{12}6^{2}$ and $5^{12}6^{4}$ cages.

Atom type	σ[ppm]	Anisotropy	Dia. cont.	Para. cont.	Cage	σ[ppm]	Anisotropy	Dia. cont.	Para. cont.	Atom type
O DAA	277.57	22.45	626.85	-349.28	$5^{12}6^{2}$	278.06	26.29	589.51	-311.45	5^{3}
	280.53	21.93	647.76	-367.23	$5^{12}6^{4}$	281.46	24.82	638.64	-357.19	
O DDA	278.93	32.20	595.44	-316.51	$5^{12}6^{2}$	278.44	28.35	632.78	-354.34	$5^{2}6^{1}$
	285.55	34.36	618.14	-332.59	$5^{12}6^{4}$	283.30	28.70	632.01	-348.70	
H DAA	23.61	20.59	355.81	-332.20	$5^{12}6^{2}$	24.71	20.30	367.27	-342.57	5^{3}
	23.84	21.24	383.51	-359.68	$5^{12}6^{4}$	24.73	21.05	377.18	-352.46	
H DAA*	30.10	12.61	-194.55	224.65	$5^{12}6^{2}$					
	30.33	12.39	-218.94	249.26	$5^{12}6^{4}$					
H DDA	25.66	19.87	387.39	-361.73	$5^{12}6^{2}$	25.22	19.93	385.44	-360.22	$5^{2}6^{1}$
	25.84	20.54	410.98	-385.14	$5^{12}6^{4}$	25.23	20.73	405.16	-379.92	

Values for monomer water, all in ppm:calculated (this work):σ_O=325.00; σ_H=31.34
exp.: σ_O=322.81[1]; σ_H=30.102[2]

Table 17: SSCCs and their components (DSO, PSO, SD and FC) for propane molecules enclathrated by $5^{12}6^2$ and $5^{12}6^4$ cages and for single propane molecule.

Coupled atoms	Molecule	J[Hz]	DSO[Hz]	PSO[Hz]	SD[Hz]	FC[Hz]	Distance[Å]
**C**H_3-**C**H_2	monomer	34.38	0.16	-0.28	1.08	33.42	1.521
	$5^{12}6^2$	34.33	0.27	-0.40	1.09	33.36	1.522
		33.54	0.27	-0.41	1.09	32.58	1.526
	$5^{12}6^4$	34.28	0.25	-0.38	1.10	33.32	1.532
		33.98	0.25	-0.39	1.09	33.03	1.532
**C**$H_3 \cdots$ **C**H_3	monomer	-0.48	-0.02	-0.23	-0.05	-0.18	2.527
	$5^{12}6^2$	-0.13	0.08	-0.28	-0.05	0.13	2.514
	$5^{12}6^4$	-0.28	0.06	-0.28	-0.05	-0.01	2.540
**CH**$_3$	monomer	131.05	0.57	1.29	-0.05	129.24	1.090
	$5^{12}6^2$	134.84	1.13	0.64	-0.07	133.14	1.093
		131.64	1.17	0.71	-0.06	129.83	1.093
	$5^{12}6^4$	135.28	1.00	0.75	-0.07	133.60	1.098
		131.36	1.02	0.86	-0.07	129.55	1.100
**CH**$_2$	monomer	130.79	0.76	1.05	-0.11	129.10	1.093
	$5^{12}6^2$	130.33	1.28	0.59	-0.11	128.58	1.097
	$5^{12}6^4$	132.56	1.15	0.63	-0.11	130.89	1.101
C**H**$_3$	monomer	-13.58	-2.75	2.92	0.40	-14.15	1.763
	$5^{12}6^2$	-14.76	-0.55	0.81	0.42	-15.43	1.762
		-12.80	-0.51	-0.80	-0.40	-13.48	1.771
	$5^{12}6^4$	-14.44	-0.91	1.14	0.41	-15.08	1.773
		-12.82	-0.91	1.16	0.40	-13.48	1.779
C**H**$_2$	monomer	-14.38	-2.31	2.45	0.45	-14.98	1.747
	$5^{12}6^2$	-13.50	-0.22	0.47	0.46	-14.21	1.753
	$5^{12}6^4$	-13.26	-0.66	0.88	0.44	-13.91	1.769

Table 18: Average values of intramolecular SSCCs of ${}^{X}J_{YZ}$ type and their components (DSO, PSO, SD and FC) for $5^{12}6^2$ and $5^{12}6^4$ cages.

Coupled atoms	J	DSO	PSO	SD	FC	Dist.	Cage	J	DSO	PSO	SD	FC	Dist.	Coupled atoms
H···H DAA	-7.73	-5.04	5.72	0.77	-9.18	1.569	$5^{12}6^2$	-8.19	-4.20	4.66	0.81	-9.46	1.559	5^3
	-7.90	-5.16	5.85	0.79	-9.38	1.560	$5^{12}6^4$	-7.44*	-4.62*	5.09*	0.75*	-8.66*	1.563*	
H···H DDA	-7.81	-4.30	4.78	0.78	-9.07	1.565	$5^{12}6^2$	-7.54	-4.37	4.87	0.76	-8.80	1.569	$5^2 6^1$
	-7.69	-4.54	5.03	0.78	-8.97	1.560	$5^{12}6^4$	-7.71	-4.53	5.03	0.78	-8.99	1.560	
O-H* DAA	-82.49	-0.28	-12.02	-0.42	-69.77	0.969	$5^{12}6^2$							
	-82.79	-0.28	-12.18	-0.45	-69. 88	0.966	$5^{12}6^4$							
O-H DAA	-82.45	-0.37	-6.88	0.07	-75.27	0.996	$5^{12}6^2$	-81.77	-0.38	-7.92	-0.05	-73.43	0.988	5^3
	-83.74	-0.36	-6.93	0.03	-76.48	0.991	$5^{12}6^4$	-84.43	-0.37	-7.74	-0.07	-76.25	0.983	
O-H DDA	-82.47	-0.38	-8.76	-0.15	-73.17	0.982	$5^{12}6^2$	-83.15	-0.38	-8.35	-0.11	-74.31	0.985	$5^2 6^1$
	-84.40	-0.37	-8.84	-0.19	-75.00	0.977	$5^{12}6^4$	-84.14	-0.37	-8.26	-0.12	-75.39	0.982	

Values for monomer (in Hz): calculated (this work): ${}^{1}J_{OH}$=-76.04, ${}^{2}J_{HH}$=-7.72
calculated (CCSD/pz3d2f)[3]: ${}^{1}J_{OH}$=-78.85, ${}^{2}J_{HH}$=-7.84
exp.: ${}^{1}J_{OH}$=-80.6[4], ${}^{2}J_{HH}$=-6.89[2]

Table 19: Average values of intermolecular SSCCs of ${}^{2h}J_{OO}$ type and their components (DSO, PSO, SD and FC) for $5^{12}6^2$ and $5^{12}6^4$ cages. Brackets contain standard deviation in % of average value.

Coupled atoms	Cage	J[Hz]	DSO[Hz]	PSO[Hz]	SD[Hz]	FC[Hz]	$r_{C\cdots O}$[Å]
DAA-DAA	$5^{12}6^2$	4.28 (19)	0.02 (4)	-0.59 (8)	0.14 (10)	4.70 (17)	2.748 (1)
	$5^{12}6^4$	4.68 (17)	0.02 (5)	-0.59 (9)	0.15 (15)	5.10 (15)	2.742 (0)
DAA-DDA	$5^{12}6^2$	2.96 (54)	0.02 (8)	-0.68 (17)	0.16 (31)	3.46 (44)	2.743 (1)
	$5^{12}6^4$	2.87 (50)	0.02 (4)	-0.69 (10)	0.16 (27)	3.38 (41)	2.745 (0)
DDA-DDA	$5^{12}6^2$	3.58 (31)	0.02 (6)	-0.76 (6)	0.21 (10)	4.11 (27)	2.752 (0)
	$5^{12}6^4$	4.20 (19)	0.02 (3)	-0.75 (4)	0.18 (6)	4.75 (16)	2.747 (0)

Table 20: Average values of intermolecular SSCCs of $^{1h}J_{OH}$ type and their components (DSO, PSO, SD and FC) for $5^{12}6^2$ and $5^{12}6^4$ cages. Brackets contain standard deviation in % of average value.

Coupled atoms	Cage	J[Hz]	DSO[Hz]	PSO[Hz]	SD[Hz]	FC[Hz]	$r_{O\cdots H}$[Å]
(O)DAA···(H)DAA	$5^{12}6^2$	7.33 (5)	-0.69 (2)	0.97 (3)	-0.41 (10)	7.46 (5)	1.763 (1)
	$5^{12}6^4$	7.27 (5)	-0.69 (2)	0.99 (2)	-0.41 (14)	7.39 (5)	1.759 (1)
(O)DAA···(H)DDA	$5^{12}6^2$	7.18 (17)	-0.68 (4)	0.92 (9)	-0.41 (16)	7.36 (15)	1.789 (2)
	$5^{12}6^4$	6.99 (15)	-0.68 (4)	0.90 (9)	-0.43 (13)	7.20 (14)	1.791 (2)
(O)DDA···(H)DAA	$5^{12}6^2$	6.76 (10)	-0.68 (2)	1.00 (2)	-0.36 (10)	6.80 (10)	1.739 (0)
	$5^{12}6^4$	6.34 (12)	-0.66 (1)	0.98 (1)	-0.37 (8)	6.40 (13)	1.746 (1)
(O)DDA···(H)DDA	$5^{12}6^2$	7.05 (6)	-0.67 (2)	0.94 (3)	-0.44 (10)	7.23 (5)	1.766 (1)
	$5^{12}6^4$	7.31 (4)	-0.68 (1)	0.94 (2)	-0.43 (5)	7.48 (4)	1.764 (0)

Table 21: Coordinates of $5^{12}6^2$ cage (in Å).

Atom	x	y	z	Atom	x	y	z	Atom	x	y	z
c1	0.945491	0.731766	0.038488	h1c1	0.630313	1.431217	-0.744091	h1c3	-1.669778	-1.520233	-0.302025
c2	0.310330	-0.634929	-0.179296	h2c1	2.032546	0.701694	0.000490	h2c3	-1.519147	-0.254756	0.936454
c3	-1.209724	-0.557788	-0.066792	h3c1	0.652876	1.145445	1.007582	h3c3	-1.596446	0.184522	-0.773228
h1c2	0.572399	-1.017211	-1.169220	h2c2	0.693600	-1.373196	0.543088				
o1	4.205877	1.013685	-0.891838	h1	3.998658	0.220189	-0.375785	h13	3.220120	-0.752761	2.174285
o2	-4.121495	1.282670	0.804605	h1a	3.643045	0.929500	-1.706767	h13a	3.161343	-0.354835	3.690268
o3	4.177287	-1.138123	0.737307	h2	-4.186520	0.483706	0.229566	h14	3.657986	-4.080981	-0.765616
o4	-4.204710	-0.969842	-0.680143	h2a	-5.023871	1.615977	0.924763	h14a	2.337405	-3.557867	-0.103778
o5	-0.965892	4.277266	0.652401	h3	5.146631	-1.111662	0.719226	h15	-3.848840	-3.747488	1.218080
o6	1.021685	-4.132721	0.889512	h3a	3.898946	-1.943596	0.231170	h15a	-2.568362	-3.521869	0.326288
o7	1.209762	4.168372	-0.941299	h4	-3.859870	-1.705303	-0.107568	h16	-3.241085	-0.755391	-2.164518
o8	-1.208597	-4.124529	-0.630681	h4a	-5.117310	-1.226777	-0.893494	h16a	-3.121559	-0.410809	-3.693347
o9	0.623141	-2.424192	3.018722	h5	-0.814526	3.786418	1.498270	h17	0.220540	-2.351142	-2.972850
o10	-2.470066	0.832728	2.967657	h5a	-0.795678	5.213455	0.835262	h17a	-1.263122	-1.763715	-2.936555
o11	-3.128600	3.319550	-0.767693	h6	0.194109	-4.076093	0.364872	h18	-0.331301	2.415995	-3.065843
o12	-0.491742	2.710112	2.872237	h6a	0.890861	-3.536527	1.659181	h18a	0.877702	3.142715	-2.348195
o13	2.601465	-0.546808	2.923301	h7	0.450474	4.013614	-0.355346	h19	2.980684	2.833528	1.387349
o14	3.132183	-3.272596	-0.664336	h7a	2.001623	3.843998	-0.435518	h19a	3.681425	2.433868	0.034959
o15	-3.269535	-3.057307	0.860967	h8	-1.030477	-3.521026	-1.432412	h20	2.067931	-0.315249	-2.800529
o16	-2.604987	-0.603840	-2.897516	h8a	-1.281956	-5.014129	-1.008106	h20a	1.824589	1.214308	-3.075616
o17	-0.726476	-2.581773	-2.866504	h9	1.307436	-1.702033	3.021336	h21	-1.105075	-1.984700	2.971569
o18	0.650657	2.485693	-3.054545	h9a	0.715108	-2.874388	3.873073	h21a	-2.442915	-2.278912	2.231581
o19	3.322219	3.198119	0.543027	h10	-2.345796	-0.147115	3.040026	h22	2.584946	-2.183961	-3.695912
o20	2.529167	0.507759	-3.023534	h10a	-3.107956	0.974505	2.240235	h22a	2.490666	-2.599258	-2.179260
o21	-2.076819	-1.890243	3.048819	h11	-3.363809	2.592601	-0.160995	h23	-2.468756	2.576954	-2.225606
o22	1.997240	-2.191629	-2.926904	h11a	-2.374123	3.766652	-0.329891	h23a	-2.082320	1.152217	-2.749003
o23	-2.073096	2.094135	-2.994099	h12	-1.152518	1.949635	2.846917	h24	1.262271	2.425956	2.808052
o24	2.208219	2.176163	2.842139	h12a	-0.650322	3.124993	3.734467	h24a	2.195521	1.213846	2.695447

Table 22: Coordinates of $5^{12}6^4$ cage (in Å).

Atom	x	y	z	Atom	x	y	z	Atom	x	y	z
c1	0.396435	0.555419	0.380896	h11	0.516079	-1.603855	0.092966	h31	-1.548505	1.562924	0.467984
c2	1.039646	-0.693596	-0.229192	h12	2.083058	-0.797406	0.087830	h32	-1.201601	0.754331	-1.087942
c3	-1.082859	0.681332	0.003119	h13	1.008682	-0.666214	-1.328636	h33	-1.641420	-0.202679	0.344861
h21	0.489104	0.527461	1.477578	h22	0.937451	1.453236	0.038326				
o1	3.051281	-2.155829	2.626312	h1a	3.484606	-1.328394	2.331891	h15a	1.819391	3.823940	-0.694232
o2	4.228821	0.127378	1.671296	h1b	2.284572	-1.890755	3.163740	h15b	1.022245	3.828579	-2.028346
o3	2.583162	-3.862950	0.520659	h2a	3.741780	0.923642	1.943725	h16a	-2.733781	-3.504326	0.973982
o4	4.525069	-0.097662	-1.054281	h2b	4.228525	0.115454	0.699174	h16b	-2.311310	-4.803230	1.735054
o5	0.9875190	-1.456907	4.305469	h3a	2.896749	-3.342695	-0.237170	h17a	0.207435	-3.220715	-3.008240
o6	2.929426	2.308381	2.730529	h3b	2.728586	-3.290199	1.308485	h17b	-0.313562	-4.025818	-1.741625
o7	3.530691	-2.563501	-1.689969	h4a	4.086713	0.553065	-1.656132	h18a	1.954868	0.753251	-3.609682
o8	0.984108	1.276447	4.350436	h4b	5.473492	0.059096	-1.145717	h18b	0.577644	0.670661	-4.334599
o9	0.004183	-4.584952	-0.107633	h5a	0.063578	-1.741223	4.052832	h19a	-2.538161	0.886728	3.527964
o10	3.226922	1.663822	-2.721363	h5b	1.166721	-1.901592	5.142751	h19b	-3.322519	0.169803	2.391784
o11	1.631277	4.069864	1.063446	h6a	2.513063	2.971785	2.138762	h20a	-1.619802	4.012271	0.876095
o12	-1.591462	-2.178909	3.677177	h6b	3.564712	2.788473	3.277907	h20b	-1.467675	4.639228	2.310531
o13	1.631865	-2.404103	-3.650149	h7a	3.966431	-1.713541	-1.463526	h21a	-3.552500	-2.529676	-0.955497
o14	-1.478835	2.286465	3.728320	h7b	4.228304	-3.147247	-2.014651	h21b	-4.663667	-3.161080	-0.077512
o15	1.927528	3.844825	-1.662130	h8a	1.704875	1.561526	3.763264	h22a	-1.308316	3.818778	-1.972234
o16	-2.059580	-3.886031	1.571525	h8b	1.013637	0.297049	4.369454	h22b	-0.831215	4.361245	-3.358496
o17	-0.572668	-3.700333	-2.636739	h9a	0.930906	-4.369190	0.165626	h23a	-2.077421	-2.848735	-2.616372
o18	1.472532	0.265236	-4.295452	h9b	-0.569924	-4.188928	0.565040	h23b	-3.500182	-2.931734	-3.262024
o19	-3.087016	0.091006	3.327204	h10a	2.796235	2.463880	-2.314160	h24a	-0.850822	2.225096	-3.583479
o20	-1.041816	4.056574	1.668491	h10b	3.679942	1.983798	-3.512957	h24b	-1.748066	1.020567	-3.968829
o21	-3.878939	-2.600339	-0.012830	h11a	0.676750	4.076342	1.316646	h25a	-4.324808	-1.055170	0.678750
o22	-0.614882	3.728549	-2.660070	h11b	1.923024	4.986575	1.161232	h25b	-5.242082	-0.215195	1.637717
o23	-3.010877	-2.427186	-2.599284	h12a	-2.159678	-1.386765	3.547508	h26a	-3.036060	3.006020	-0.764240
o24	-0.965677	1.538384	-4.257997	h12b	-1.762307	-2.788905	2.939075	h26b	-3.102369	4.553672	-0.782914
o25	-4.525934	-0.142660	0.994375	h13a	1.542246	-1.466601	-3.930135	h27a	-3.345294	0.637913	-2.376505
o26	-2.480735	3.822910	-0.664338	h13b	2.306553	-2.409713	-2.950127	h27b	-3.035120	-0.710392	-3.071256
o27	-3.170210	0.242154	-3.244587	h14a	-1.268707	2.653829	2.857963	h28a	-4.293258	1.040566	-0.295867
o28	-4.088915	1.627451	-1.065454	h14b	-0.609644	1.926071	4.039364	h28b	-4.932201	1.771486	-1.515404

[1] A. Antusek, K. Jackowski, M. Jaszunski, W. Makulski, M. Wilczek, Nuclear magnetic dipole moments from NMR spectra, Chemical Physics Letters 411 (1-3) (2005) 111 – 116. doi:DOI:10.1016/j.cplett.2005.06.022.
URL http://www.sciencedirect.com/science/article/B6TFN-4GH49XS-7/2/d7c972b82dc0b38eb450cd4139b9ed94

[2] W. Makulski, An ^{2}H(D) isotope shift in the ^{1}H NMR spectra of water in gaseous environment of fluoromethanes, Journal of Molecular Structure 839 (1-3) (2007) 90–93. doi:10.1016/j.molstruc.2006.10.047.
URL http://linkinghub.elsevier.com/retrieve/pii/S0022286006008714

[3] A. A. Auer, J. Gauss, Triple excitation effects in coupled-cluster calculations of indirect spinspin coupling constants, The Journal of Chemical Physics 115 (4) (2001) 1619. doi:10.1063/1.1386698.
URL http://link.aip.org/link/JCPSA6/v115/i4/p1619/s1&Agg=doi

[4] N. M. Sergeyev, N. D. Sergeyeva, Y. A. Strelenko, W. T. Raynes, The ^{1}H—^{2}H, ^{17}O—^{1}H coupling constants and the ^{16}O/^{18}O induced proton isotope shift in water, Chemical Physics Letters 277 (1-3) (1997) 142 – 146. doi:DOI:10.1016/S0009-2614(97)00933-0.
URL http://www.sciencedirect.com/science/article/B6TFN-3SDCFKT-T/2/0ad583fe903abc6d2a340eb22a77766c
